# Lactic Acidosis Interferes With Toxicity of Perifosine to Colorectal Cancer Spheroids: Multimodal Imaging Analysis

**DOI:** 10.3389/fonc.2020.581365

**Published:** 2020-12-04

**Authors:** Barbora Pavlatovská, Markéta Machálková, Petra Brisudová, Adam Pruška, Karel Štěpka, Jan Michálek, Tereza Nečasová, Petr Beneš, Jan Šmarda, Jan Preisler, Michal Kozubek, Jarmila Navrátilová

**Affiliations:** ^1^ Department of Experimental Biology, Faculty of Science, Masaryk University, Brno, Czechia; ^2^ Department of Chemistry, Faculty of Science, Masaryk University, Brno, Czechia; ^3^ Department of Chemistry and Applied Biosciences, ETH Zurich, Zurich, Switzerland; ^4^ Centre for Biomedical Image Analysis, Faculty of Informatics, Masaryk University, Brno, Czechia; ^5^ Center for Biological and Cellular Engineering, International Clinical Research Center, St. Anne’s University Hospital, Brno, Czechia

**Keywords:** perifosine, Akt kinase, tumor environment, lactic acidosis, alkalization, signal co-registration, mass spectrometry imaging, colorectal cancer

## Abstract

Colorectal cancer (CRC) is a disease with constantly increasing incidence and high mortality. The treatment efficacy could be curtailed by drug resistance resulting from poor drug penetration into tumor tissue and the tumor-specific microenvironment, such as hypoxia and acidosis. Furthermore, CRC tumors can be exposed to different pH depending on the position in the intestinal tract. CRC tumors often share upregulation of the Akt signaling pathway. In this study, we investigated the role of external pH in control of cytotoxicity of perifosine, the Akt signaling pathway inhibitor, to CRC cells using 2D and 3D tumor models. In 3D settings, we employed an innovative strategy for simultaneous detection of spatial drug distribution and biological markers of proliferation/apoptosis using a combination of mass spectrometry imaging and immunohistochemistry. In 3D conditions, low and heterogeneous penetration of perifosine into the inner parts of the spheroids was observed. The depth of penetration depended on the treatment duration but not on the external pH. However, pH alteration in the tumor microenvironment affected the distribution of proliferation- and apoptosis-specific markers in the perifosine-treated spheroid. Accurate co-registration of perifosine distribution and biological response in the same spheroid section revealed dynamic changes in apoptotic and proliferative markers occurring not only in the perifosine-exposed cells, but also in the perifosine-free regions. Cytotoxicity of perifosine to both 2D and 3D cultures decreased in an acidic environment below pH 6.7. External pH affects cytotoxicity of the other Akt inhibitor, MK-2206, in a similar way. Our innovative approach for accurate determination of drug efficiency in 3D tumor tissue revealed that cytotoxicity of Akt inhibitors to CRC cells is strongly dependent on pH of the tumor microenvironment. Therefore, the effect of pH should be considered during the design and pre-clinical/clinical testing of the Akt-targeted cancer therapy.

## Introduction

Colorectal carcinoma (CRC) is the fourth most common cause of cancer death in Western countries (1, 2). More than 1.8 million new cases of CRC were diagnosed in 2018 (3). Accumulation of several genetic mutations including those affecting the PI3K/Akt pathway has been described during CRC development (1, 4–6). Akt transduces signals regulating multiple biological processes, such as cell proliferation, survival, growth, angiogenesis, migration and epithelial-mesenchymal transition in CRC as well as other cancers (6, 7). Therefore, targeting Akt has been suggested as a potential therapeutic strategy in cancer diseases (1, 5, 6, 8).

One of the most promising Akt inhibitors, perifosine, is a synthetic alkyl phospholipid that targets signal transduction pathways at the cell membrane by preventing correct localization of the Akt kinase, thus precluding its phosphorylation (9–12). Perifosine was shown to effectively target the PI3K/Akt pathway both in preclinical models and in clinical trials (10, 11). However, clinical studies resulted in disappointing response rates of common solid tumors to perifosine as a single agent, while combination with another therapies seemed to be more effective (13–15).

One of the factors contributing to the late recognition of insufficient cytotoxicity of drugs, occurring often in up to the third phase of clinical trials, is the model used for *in vitro* tests. Although it is well known that cells cultured in monolayer do not mimic the real environment, they are still widely used in preclinical research (16). Model dimensionality directly affects features of tumor-specific environment and as a result cell proliferation, differentiation, protein expression and response to cellular stimuli. *In vivo*, real tumors exhibit also increased expression of extracellular matrix proteins, increased stiffness, more natural cell-to-cell contacts, intersticial flow, stromal and immune cells, and gradients of nutrients, oxygen and pH. The efforts to define pH of the *in vivo* growing tumor (17) and manipulations of extracellular pH to support therapy have been performed on 2D and 3D models in the past (18–22). Moreover, the intercapillary distance is relatively large in tumor tissues, and this contributes to reduced transport of drugs. For these reasons, the 3D tumor models are considered to be more suitable for drug testing (16, 23–28). Acquisition of drug resistance followed by chemotherapy failure is frequently associated with establishment of acidic tumor-specific microenvironment (19, 29–33). Interestingly, tumor alkalization may restore drug efficacy and reduce cancer invasion (29, 34). Extracellular acidosis might result from dysregulated tumor metabolism, increased glycolytic activity or poor vascular perfusion (35, 36). As a result, the pH of the tumor microenvironment may reach the values ranging from 6.2 to 6.9. Furthermore, colorectal tumors could be exposed to a wide range of intraluminal pH (5.9 – 8.1) depending on their localization in the intestinal tract, patient diet, and activity of intestinal microflora (37–41). This fact is mostly overlooked in CRC research.

Limited drug penetration into tumor tissue might lead to chemotherapy failure and drug resistance. The matrix-assisted laser desorption/ionization time-of-flight mass spectrometry imaging (MALDI TOF MSI) has been used to localize the distribution of drugs in colon, ovarian, breast or pancreatic tissue (42–45). Cell apoptosis and proliferation markers can be detected by immunohistochemistry (IHC) (46, 47). Since the 3D models emerge as a valuable tool for high-throughput drug screening and nanomedicine research (23, 48, 49), an efficient workflow for spatial co-localization of drug distribution with IHC-detectable biological response is essential for the correct interpretation of the results. Therefore, we have recently introduced the IHC-compatible MALDI mass spectrometry (MS) protocol employing fiducial markers for co-localization of signals. Quantification of MS and IHC signals in equidistant layers from the spheroid boundary to its center has been used to obtain information about the effects of drugs on cell proliferation, apoptosis, metabolism and other processes (50). Since the MALDI MSI analysis setting does not modify the protein epitopes (51), MS and IHC can be performed on the same spheroid section.

This work aims to clarify the effect of specific features of the CRC tumor and colon microenvironment on perifosine distribution and cytotoxicity. Using 2D and 3D CRC models, we observed that perifosine cytotoxicity was strongly pH dependent. Perifosine penetration into cells was pH-dependent in 2D but was rather low and not influenced by extracellular pH in 3D settings.

## Methods

### Cell Cultures and Chemicals

HT-29 and HCT-116 human adenocarcinoma cells (ATCC®, HTB-38™) were obtained from LGC Standards (Teddington, UK). Cells were cultivated in humidified 5% CO_2_ at 37°C in DMEM (Sigma-Aldrich, St. Louis, Missouri, USA) with 10% fetal bovine serum (FBS) (Invitrogen, Paisley, UK), 2 mM L-glutamine, 100 U/mL penicillin and 100 U/mL streptomycin (Lonza, Basel, Switzerland). Adherent cells were harvested with trypsin (Biosera, Nuaillé, France) and counted using the Bürker chamber (Assistent, Sondheim vor der Rhön, Germany). The cells were treated with a stock solution of 5 mM perifosine (Sigma-Aldrich, St. Louis, Missouri, USA) in 50% ethanol (VWR International, Monroeville, Pennsylvania, USA).

### pH of Culture Media

Conditions of eight different culture media mimicking the tumor microenvironment (normal pH/normoxia, acidic pH/normoxia, normal pH/hypoxia, acidic pH/hypoxia and each of them with alkalization) were simulated by pH modulation of RPMI 1640 media (Sigma-Aldrich, St. Louis, Missouri, USA) by lactic acid (LA) and sodium bicarbonate (NaHCO_3_). As a control, normal pH 7.4 was maintained by the addition of 20 mM sodium lactate (NaL) excluding the effect of lactate alone on drug efficacy (52). The pH of the culture media was determined by the HydroDish® HD24 (PreSens, Freiburg, Germany) at 37°C, 5% CO_2_ after 24 h in wells without the cells (**Table 1**). These pH values of conditioned media were used for description of all experiments. For hypoxic cell culture (1% O_2_), the BioSpherix I-Glove cell culture chamber (BioSpherix, New York, USA) was used. Furthermore, the presence of hypoxic conditions (1% O_2_) in this chamber was verified by the optical oxygenic probe WTW-FDO 925, WTW Multi 3410 (WTW—measuring and analytical technology, Prague, Czechia). The up-regulation of carbonic anhydrase IX level finally proved the exposition of cells to hypoxia (not shown).

**Table 1:** pH of the modified culture media.

The environment	Abbreviation	Concentration of chemicals	pH
Lactic acid	LA	20 mM	6.6
Lactic acid with sodium bicarbonate	LA + NaHCO_3_	20 + 20 mM	7.3
Sodium lactate	NaL	20 mM	7.4
Sodium lactate with sodium bicarbonate	NaL + NaHCO_3_	20 + 20 mM	7.9
Sodium bicarbonate	NaHCO_3_	80 mM	8.1

The culture media were prepared by pH alteration of RPMI media using 20 mM lactic acid to decrease the pH or 20 mM/80 mM NaHCO3 to increase the pH. As a control, 20 mM sodium lactate was used. The pH was measured by the HydroDish® HD24 at 37°C, 5% CO2 after 24 h in wells without the cells.

To determine alterations of pH during cultivation of cells grown in monolayers, we monitored pH in real-time using the HydroDish® HD24 plates. The cells (2×10^5^ cells/mL) were cultivated in media supplemented with NaL, LA and NaHCO_3_ in concentrations described in **Table 1**. The experiments were performed in normoxia and hypoxia for 48 h.

### Three-Dimensional Cell Culture Models

For multicellular spheroid formation, the HT-29 and HCT-116 cells were seeded at a density of 5×10^4^ cells/mL on the agar-coated (1% agar in 1× PBS) 12-well plate (Costar®, Corning Incorporate, Kennebunk, Maine, USA) in DMEM supplemented with 2 mM L-glutamine and lacking FBS. Cells were incubated on a rotary shaker (Orbital Shaker, NB-101SRC, N-BIOTEK, Korea) at 75 rpm in a humidified 5% CO_2_ atmosphere at 37°C for 3 h, after which FBS was added at a 1% concentration. The next day, FBS was supplied to a final concentration of 10%, and the spheroids were cultivated for another 5 days at 60 rpm (51).

### Cell Proliferation and Viability

#### MTT Assay

Cell metabolic activity was measured using the MTT assay. Briefly, cell suspension (2×10^5^ cells/mL) was seeded to the 24-well plates (Costar®, Corning Incorporate, Kennebunk, Maine, USA) and cells were pre-treated in culture media with various pH (pH 6.6, 7.3, 7.4, and 7.9) for 72 h. Next, the depleted media were replaced with fresh ones, and the cells were treated with perifosine (final concentration 10 and 20 µM). Control cells were treated with 50% ethanol as a solvent. The medium was removed, and 500 µL of 10% MTT (5 mg/mL) in RPMI was added for 1 h. Then, harvested cells were centrifuged, the supernatant removed, and 150 µL of dimethyl sulfoxide (DMSO) was added to dissolve formazan crystals. Optical density was measured as a difference at two wavelengths (570–650 nm). For the MTT assay on 3D models, spheroids were treated with 20 µM perifosine or 50% ethanol in different culture media (pH 6.6, 7.4 for 72 h, pH 8.1 for 48 h). Spheroids were loosened by trypsin, pellets were collected by centrifugation and 200 µL of 10% MTT (5 mg/mL) in RPMI medium was added for 2.5 h. Optical density was determined as described for the 2D cell culture.

#### ATP Assay

The cells were treated with perifosine as described for the MTT assay. Quantification of cellular ATP was performed in cells cultured in 24-well plates using the ATP assay kit (Cayman Chemicals, Ann Arbor, Michigan, USA) as described earlier (53). The ATP concentration was subtracted from the calibration curve. The 3D models were disintegrated by trypsin and subjected to ATP assay using the same procedure.

#### Quantification of the Cell Cycle by Flow Cytometry

The cells (2×10^5^ cells/mL) were seeded to the 6-wells plates (Costar®, Corning Incorporate, Kennebunk, Maine, USA) and left in various culture media for three days. Next, the depleted media were replaced with fresh ones and the cells were treated with perifosine (20 µM) for 24 h and harvested using trypsin. For detection of cells with fragmented DNA, the samples were processed by the citrate-modified cell cycle analysis, using flow cytometry (FACSVerse, BD, Andover, Massachusetts, USA) as described previously (54). The cell populations were quantified using FACSuite software (BD, Andover, Massachusetts, USA).

#### Live/Dead Cells Staining and LSCM Analysis

Viability of cells up to 100 µm from the spheroid surface was determined using the calcein-AM/propidium iodide live/dead cells staining. Experiments were performed in 12-well plates. Spheroids were treated with perifosine in different culture media (pH 6.6 and 7.4 for 72 h, pH 8.1 for 48 h). Next, 1 µM calcein-AM (ChemCruz, Santa Cruz Biotechnology, Santa Cruz, California, USA) and 3 µM propidium iodide (Cayman Pharma, Neratovice, Czech Republic) were added for 1 h. Subsequently, spheroids were washed in 1×PBS, and emitted fluorescence was detected by laser scanning confocal microscopy (LSCM) (TCS SP8, Leica Microsystems, Wetzlar, Germany). The wavelength for the calcein excitation was 488 nm, and the detection window was set to 500–550 nm. The propidium iodide was excited with 552 nm laser, and the emission was detected in the range of 590–670 nm. Cell viability was finally determined as the ratio of dead and live cells using TissueQuest software (TissueGnostics, Wien, Austria).

### Determination of pHe Using the SNARF-5F Probe

For determination of the extracellular pH up to 15 µm from the spheroid surface (pHe), the cell-impermeant pH indicator, carboxy SNARF®-5F dye (S23922, Thermo Fisher Scientific, Waltham, Massachusetts, USA) was used. For calibration data, RPMI medium alone or supplemented with either LA or NaHCO_3_ was prepared. pH values in these media were determined by HydroDish® HD24 plates (PreSens, Regensburg, Germany) in 37 °C, 5% CO_2_. Then, the SNARF-5F dye was added into calibration media (22 µM final concentration). Fluorescence emissions of this probe at 520–580 and 630–700 nm were simultaneously recorded in two channels of the confocal microscope in response to 488-nm laser excitation. Ratios of these fluorescence signals were calculated for each pH and plotted against the respective pH. All fluorescence measurements were performed at 37°C, 5% CO_2_. Fluorescence images of the samples were acquired using Leica Application Suite X (LAS X) software (Leica Microsystems, Wetzlar, Germany). For pHe determination, the spheroids were exposed to the cultivation media with or without LA (pH 6.6) or NaHCO_3_ (pH 8.1) for three days. Then, the spheroids were loaded with SNARF®-5F dye (22 µM final concentration) for 4 h, and emitted fluorescence was measured. To eliminate autofluorescence, the spheroid without SNARF-5F loading was used as a control.

### Gel Electrophoresis and Immunoblotting

Cells (2×10^5^ cells/mL) were seeded in various culture media for 72 h. Then, the depleted media were replaced with fresh ones, and the cells were treated with perifosine for 24 and 48 h or left untreated. The cells were collected, lysed, and processed for gel electrophoresis followed by immunoblotting as described (55). The blots were probed with phospho-Akt, total Akt-, cleaved caspase 8-, cleaved PARP- (CST4060, CST4691, CST9496, CST5625, Cell Signaling Technology, Beverly, Massachusetts, USA) and α-tubulin-specific (ab7291, Abcam, Cambridge, UK) antibodies. For developing, goat anti-rabbit or anti-mouse secondary antibody conjugated to peroxidase (Cell Signaling Technology, Beverly, Massachusetts, USA) and standard ECL procedure with Immobilon Western Chemiluminescent HRP Substrate (Millipore, Burlington, Billerica, Massachusetts, USA) were used.

### Treatment of Spheroids in the Floating Media

Spheroids were transferred to the 12-well plate with fresh RPMI media and treated with 20 µM perifosine for 6, 24, 48, and 72 h to determine perifosine penetration. To analyze the effect of microenvironment on perifosine penetration, the spheroids were treated with 20 µM perifosine in media with pH adjusted to either 6.6 or 8.1 for 72 and 48 h, respectively. As a control, the standard culture medium with pH 7.4 was used. Finally, all the spheroids were collected in cryomolds with warm gelatine and frozen at −80°C. Next, the sectioned tissue was processed for the perifosine analysis (MALDI MS) followed by cleaved caspase 8 determination (IHC) (briefly described in the **Supplementary Data Sheet 1**), full description in (51). Unless otherwise stated, experiments were performed under floating conditions.

### Treatment of Spheroids in the Collagen Matrix

Collagen was used for the culture of epithelial cancer cells in 3D setting. Compared to Matrigel, its composition is well defined, can be used in translational medicine and is easy to prepare (56). To better mimic *in vivo* conditions, glycated collagen has been used (57). Collagen glycation results in its stiffening, significantly altering its biological properties and was previously associated with cancer progression (57, 58).

For matrix preparation, collagen I (Sigma-Aldrich, St. Louis, Missouri, USA) was non-enzymatically glycosylated with glucose (Sigma-Aldrich, St. Louis, Missouri, USA, final concentration 100 mM) for five days at 4°C and then mixed with 3× concentrated cultivation medium in a 2:1 ratio (59). The pH was set to 7 or 8 using 1 M NaOH. The spheroids were embedded in 300 µL of this matrix and left at 37°C until solidified, then covered by 50 µL of culture medium and treated with perifosine for 48 h. Subsequently, the medium was removed, whole collagen matrix blocks were fixed by 4% paraformaldehyde for 24 h and dehydrated in 30% solution of sucrose (Sigma-Aldrich, St. Louis, Missouri, USA) for 7 days. Blocks were transferred to warm gelatine and frozen at −80°C. Finally, the spheroids were sectioned and subjected to MALDI MS and IHC analyses.

### MALDI TOF MS of 2D and 3D Cell Cultures

MALDI MSI settings and the workflow for detection of the perifosine spatial distribution in 3D were fully described by Machálková et al., 2019 (51). For an overview, see the **Supplementary Data Sheet 1**, Methodology.

#### Perifosine Detection in Cell Culture Monolayers

Cells (2×10^5^ cells/mL) cultivated in different tumor environments for 72 h were treated with perifosine (20 µM) for 2 h. Then, 6×10^5^ cells in 100 µl of 1×PBS was cytocentrifuged (Rotofix 32A, HETTICH Zentrifugen, Tuttlingen, Germany, 1,000 rpm/5 min) on 4 positions of an indium tin oxide (ITO) conductive glass slide. The diameter of each spot was approximately 5 mm. The samples were covered by sublimated 2,5-dihydroxybenzoic acid (DHB, 0.24 mg/cm^2^) and dried in a desiccator for at least 30 min. Perifosine in spots of cytocentrifuged cells was measured using MALDI MSI (see the **Supplementary Data Sheet 1**, Methodology). However, since the imaging mode was not employed to obtain spatial information, but rather to automatically measure selected areas of all 4 spots at once, the laser beam diameter was set to a relatively large value of 70–80 µm and the pixel size was 200 µm. To obtain maximum information from the 200-µm pixel area, a total number of 1,000 laser shots were summed within the pixel, with random movement of the laser firing 200 shots/position. The number of pixels measured from each cytocentrifuged spot was approximately 40 to control the pixel-to-pixel reproducibility. The cytocentrifugation experiment was performed in duplicates the same day (2 glass slides) and in triplicates during a longer period.

#### Spatial Distribution, Co-Localization, and Quantification of MALDI MSI and IHC Signal in Spheroid Sections

The signals of perifosine from MALDI MSI and antibodies specific for Ki-67 and cleaved caspase 8 from IHC were obtained. The IHC protocol is described in **Supplementary Data Sheet 1**. The signals were co-localized and quantified. Then, the respective intensities of both MS and IHC modalities were evaluated along equidistant layers (“peels”) starting from the spheroid boundary. Since sections may contain areas with no tissue and therefore no meaningful signal, we used interactive thresholding to exclude these gaps from further analysis. The same threshold was used for all samples to be compared. Semiautomatic co-registration of MALDI MSI and IHC signals and the “peeling” analysis were also described by our group recently (50, 51).

### Statistical Data Analysis

Distributions of the collected data were described in boxplots, showing medians (a thick mark), interquartile range (box bounding) and range (the whiskers of boxplots correspond to the minimum and maximum values). Statistical comparison for viability assays was performed using a two-sample t-test. In the case of non-normal distribution of data (tested via Shapiro-Wilk test), a log transformation was applied before the test. All experiments were performed at least in triplicate. For statistical evaluation of signal co-registration, the protocol presented in (51) was followed. The conclusions drawn from MALDI MS and IHC co-registration were based on at least three independent observations.

## Results

### Acidosis Decreases the Intracellular Level and Cytotoxicity of Perifosine on CRC Cells in Monolayers

**Figure 1:**
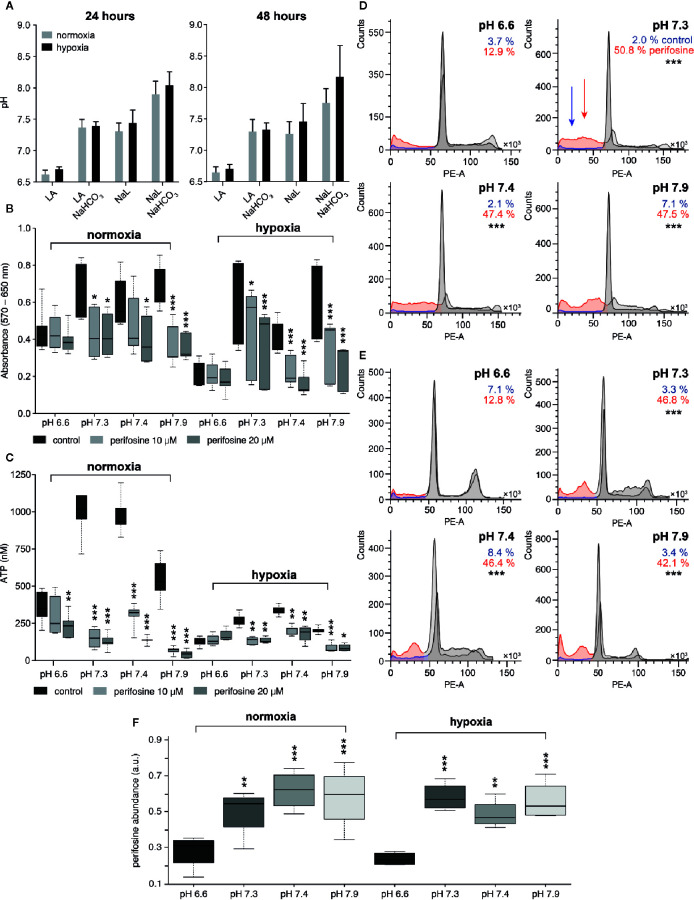
The effect of the tumor-specific environment on perifosine cytotoxicity in 2D cell cultures. **(A)** The pH of conditioned media in normoxia and hypoxia; acidic media (LA), control conditions (NaL), alkalized media (LA+NaHCO_3_ and NaL+NaHCO_3_). Results are presented as an average with standard deviation. **(B, C)** The effect of the pH on the perifosine cytotoxicity evaluated by MTT and ATP assays. Significant difference (*) in both absorbance and ATP value between controls and perifosine-induced samples is shown. **(D, E)** The frequency (%) of the cells in the subG0/G1 phase of the cell cycle in **(D)** normoxia and **(E)** hypoxia; control (blue) and perifosine-treated cells (red); statistically significant difference (*) between the cells treated in acidosis and other conditions is shown. **(F)** The level of perifosine determined in the cells cultivated in various tumor-specific environments using MALDI MSI; the significant increase of perifosine abundance between acidic (pH 6.6) and other conditions (*) are highlighted. Results are presented in boxplots showing median, interquartile range, minimum and maximum values; significant difference of all experiments was evaluated by t-test; *p < 0.05, **p < 0.01, ***p < 0.001.

Cytotoxicity of drugs used to eliminate tumor cells *in vivo* can be significantly affected by specific features of the tumor, especially by acidosis and hypoxia. Therefore, *in vitro* culture conditions were optimized to mimic the corresponding values of tumor pH and hypoxia *in vivo*. The pH of cell culture media in the wells during cell cultivation was monitored by the PreSens method. After 24 h of cultivation in normoxia, the pH of the original culture medium and NaL-enriched medium was 7.4, while the pH of the LA-supplemented medium was 6.6. The decrease of pH induced by LA could be reversed by NaHCO_3_ (20 mM) back to the normal pH value 7.3. On the contrary, in control wells with NaL, addition of NaHCO_3_ resulted in an increase of pH to 7.9 (**Figure 1A**). After 48 h, slight acidification of the NaL- and NaHCO_3_-supplemented medium was detected, presumably due to cellular metabolism (**Figure 1A**). These results verified that the cells in our cultures were exposed to physiological pH values within the range of those found either in the intestinal tract or in the extracellular space of tumors.

Then, we investigated the effect of extracellular acidosis and hypoxia on cytotoxicity of perifosine to HT-29 cells. We found that cytotoxicity of perifosine used in clinically relevant 10 and 20 µM concentrations (60, 61) for 24 h was suppressed by both acidosis alone and acidosis associated with hypoxia, as assessed by the MTT and ATP assays (**Figures 1B, C**). Less severe acidosis (pH 6.8 and higher) was not detrimental to perifosine efficacy (**Supplementary Image 1A**). Both controls and perifosine-induced cells exposed to acidic conditions pH 6.6 were still live and metabolically active, as verified by strong decrease of their metabolic activity after inhibition of glucose uptake and oxidative phosphorylation (OXPHOS) (**Supplementary Image 1B**). Addition of NaHCO_3_ to the medium supplemented with LA (pH 7.3) restored perifosine efficacy, both in normoxia and hypoxia (**Figures 1B, C**). No difference in perifosine cytotoxicity was observed in the medium alone and the medium supplemented with NaL (**Supplementary Image 2**). Further alkalization of the NaL medium to a value 7.9 did not improve perifosine efficacy (**Figures 1B, C**). To confirm that the effect of media pH on cytotoxicity of perifosine is not cell line specific, we performed similar experiments using HCT-116 cells. We confirmed that cytotoxicity of perifosine on these cells was reduced by acidosis as well, although to a lesser extent than to HT-29 cells. Perifosine efficacy to HCT-116 cells exposed to hypoxia was affected by the fact that their survival in hypoxic conditions is limited (**Supplementary Image 3**). The importance of the pH for perifosine cytotoxicity was verified by the cell cycle analysis (**Figures 1D, E**). Frequency of the perifosine-treated cells with fragmented (subG0/G1) DNA presumably undergoing apoptosis significantly differed in acidic (pH 6.6) and other conditions (pH 7.3, 7.4, 7.9), both in normoxia and hypoxia (**Figures 1D, E**, **Supplementary Table 1**). Again, we verified that there was no difference in the perifosine cytotoxicity in media containing or lacking NaL. This indicates that lactate alone did not affect cytotoxicity of perifosine (data not shown). For complete information on the frequency of the cells detected in all phases of the cell cycle see **Supplementary Table 1**.

To confirm the role of pH in regulation of the perifosine ability to induce apoptosis, intracellular levels of cleaved PARP and caspase 8 were determined by immunoblotting. Compared to acidic conditions, elevated levels of cleaved PARP and cleaved caspase 8 were detected in HT-29 cells exposed to perifosine at pH 7.4 and normoxia. Alkalization of the LA-containing medium increased the levels of cleaved PARP and caspase 8 after treatment with perifosine (**Supplementary Image 4**). To confirm that perifosine inhibits the Akt activity under these conditions, the level of phospho-Akt (Ser473) was also determined. The decrease of the phospho-Akt protein was detected in normal, acidic and alkaline conditions. Similar results were obtained for the cells cultivated in hypoxia (**Supplementary Image 4**).

Since acidosis can limit the uptake of drugs by cells, we assessed the intracellular level of perifosine in HT-29 cells exposed to normoxia/hypoxia combined with either normal conditions or acidosis by MALDI MSI. Suitability of MALDI MSI for detection of perifosine was verified by laser capture microdissection followed by offline liquid extraction coupled to liquid chromatography with electrospray ionization mass spectrometry (51). Interestingly, the intracellular level of perifosine in cells exposed to acidosis was lower than in cells cultivated in normal pH (**Figure 1F**). This effect was reverted by alkalization of the culture medium up to 7.3 or 7.9 (**Figure 1F**). A similar effect of pH on the intracellular level of perifosine was found in hypoxia (**Figure 1F**).

### Perifosine Cytotoxicity and Spreading Through the Spheroids Are Limited

To test perifosine cytotoxicity in the 3D models, spheroids of HT-29 cells were treated with perifosine for 48 or 72 h. Cytotoxicity was assessed by the MTT assay performed either on intact or the trypsin-dissociated spheroids (**Supplementary Image 5**). Compared to the trypsin-dissociated spheroids, the intact spheroids exhibited only a minor decrease of viability after treatment with perifosine. This results from poor penetration of MTT into the spheroids, as verified by the bright-field microscopy of the spheroid sections (**Supplementary Image 5**). Therefore, for further experiments, the spheroids were dissociated into a suspension of individual cells before starting the MTT assays.

**Figure 2:**
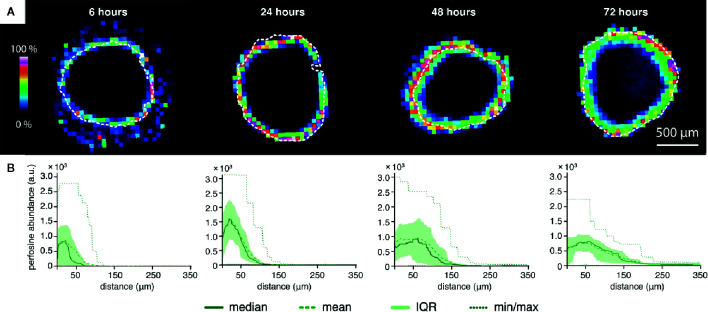
The time-dependent peeling analysis of the perifosine penetration into the spheroids obtained by MALDI MSI. **(A)** MALDI MSI of perifosine distribution in the spheroid sections during the 6, 24, 48, and 72 h of treatment co-localized with the spheroid boundary detected by optical microscopy (white dashed line). **(B)** The distribution of perifosine abundance across the respective distances. The median and mean abundance in the peels are represented by the solid and dashed lines, respectively. Minimum and maximum values are represented by the dotted lines, the area of the interquartile range (IQR, i.e. values between the 25th and 75th quantiles) is highlighted in green. The zero at the X-axis coincides with the outer edge of the spheroid, while the Y-axis shows the peel abundance of perifosine.

Next, we analyzed the perifosine distribution in the spheroids treated with perifosine for 6, 24, 48, and 72 h using MALDI MSI (**Figure 2**). The MS images of perifosine spots inside the spheroid and their quantification by peeling analysis are shown in **Figures 2A, B**. The MS imaging of the perifosine distribution revealed some perifosine signals in the gelatine surrounding the spheroid boundary (**Figure 2**). As reported previously, this perifosine signal can be explained by increased perifosine ionization in gelatine compared to biological tissue (51). Therefore, only perifosine signal within the spheroid section was quantified by peeling analysis. After 6 h, perifosine penetrated approximately 50 µm from the spheroid surface. During incubation prolonged to 72 h, perifosine penetrated up to the maximum distance of 200 µm. The statistical analysis of the MS signal revealed a very heterogeneous distribution of perifosine within spheroids, not only from the spheroid periphery to its central part but also within the same peel—within the same distance from the spheroid boundary (**Figure 2B**).

**Figure 3:**
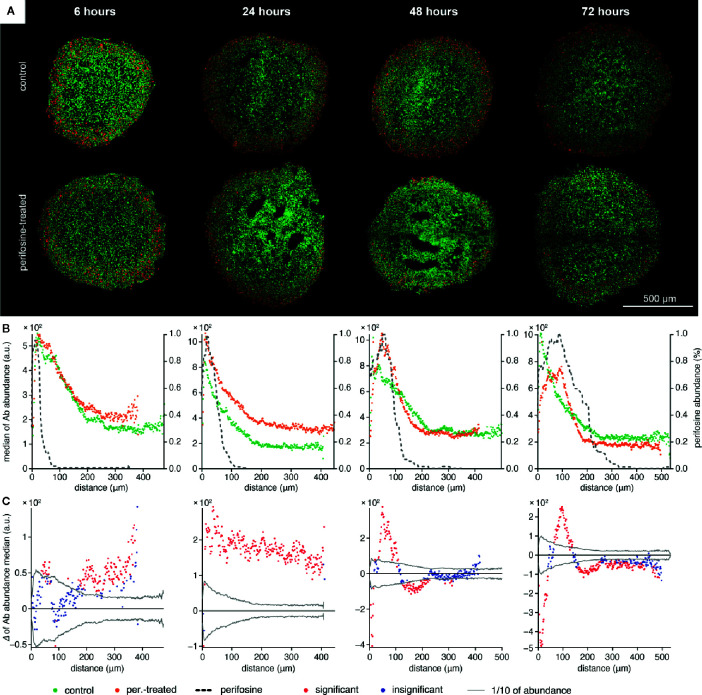
The time-dependent co-localization of the perifosine and Ki-67 spatial distribution in the spheroids. **(A)** IHC images showing the signal of Ki-67 (red) and nuclei (green) for control and perifosine-treated (20 µM) spheroids. **(B)** Peeling analysis of Ki-67 and perifosine distributions within the spheroid sections. Distribution of the Ki-67 (median per peel) for control (•) and perifosine-treated (•) spheroids are depicted. The perifosine signal was re-scaled using min-max normalization and its distribution is marked by the dashed line. **(C)** Differences between the median values of Ki-67 abundance in the control and the perifosine-treated spheroids. The median value of the control spheroid was subtracted from the median value of the induced spheroid in each peel. The zero at the X-axis coincides with the outer edge of the spheroid. The significant (•) and insignificant (•) differences of medians for p < 0.05 are distinguished.

**Figure 4:**
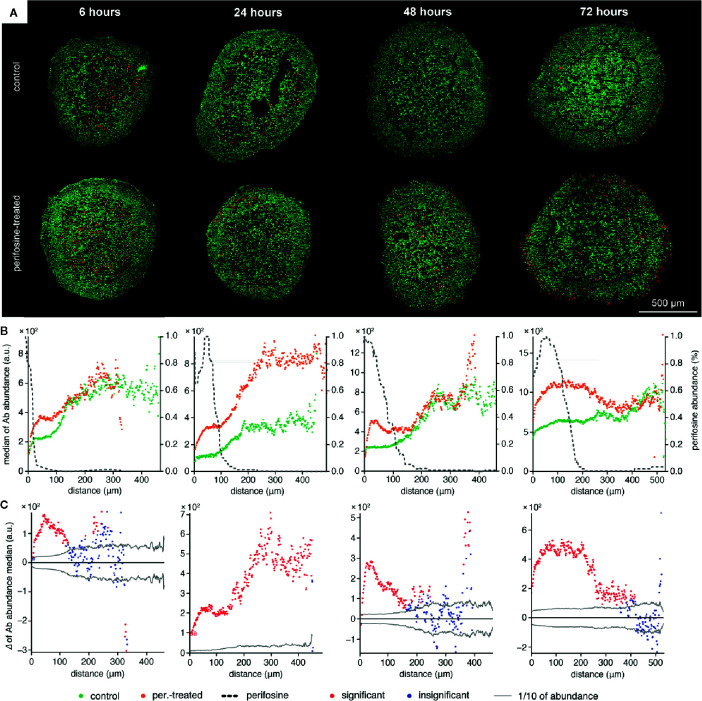
The time-dependent co-localization of the perifosine and the cleaved caspase 8 spatial distribution. **(A)** IHC images showing the signal of cleaved caspase 8 (red) and nuclei (green) for control and perifosine-treated (20 μM) spheroids. **(B)** Peeling analysis of cleaved caspase 8 and perifosine distributions within the spheroid sections. Distribution of the cleaved caspase 8 (median per peel) for control (•) and perifosine-treated (•) spheroids are depicted. The perifosine signal was re-scaled using min-max normalization, and its distribution is marked by the dashed line. **(C)** Differences between the median values of cleaved caspase 8 abundance in the control and the perifosine-treated spheroids. The median value of the control spheroid was subtracted from the median value of the induced spheroid in each peel. The zero at the X-axis coincides with the outer edge of the spheroid. The significant (•) and insignificant (•) differences of medians for p < 0.05 are distinguished.

To investigate the effect of perifosine on the biological response of the cells forming the spheroids, we co-localized its spatial distribution with markers of proliferation and apoptosis detected by IHC. Using the protocol for the co-localization of the MALDI MSI and fluorescence IHC data (50, 51), the distributions of Ki-67 (**Figure 3**) and cleaved caspase 8 (**Figure 4**) were examined. The distribution of Ki-67 and cleaved caspase 8 obtained by LSCM is depicted in **Figures 3A** and **4A** respectively, and the resulting graphs of both proteins and perifosine signals median values from the spheroid boundary towards its center are shown in **Figures 3B** and **4B**. The differences between median values of IHC signal in the control and the perifosine-induced spheroids are shown in **Figures 3C** and **4C**. This workflow allowed us to make the following conclusions for Ki-67 distribution. First, in each time interval, independently of perifosine induction, the highest abundance of the Ki-67 signal appeared within 200 µm from the spheroid boundary with a strong decline toward the spheroid center (**Figures 3A, B**). Second, analysis of differences between the Ki-67 median values in control and the perifosine-treated spheroids (**Figure 3C**) revealed increase of the Ki-67 abundance in the perifosine-free regions of the spheroids treated with the drug for 6 and 24 h. In the 24 h interval, the Ki-67 signal was stronger in the entire induced spheroid section, while in 48 and 72 h intervals, an abundance of Ki-67 diminished in the perifosine-exposed regions within the distance of 50 µm from their boundary. This region of Ki-67 down-regulation adjoined to the area of 50–100 µm from the spheroid boundary with elevated Ki-67 abundance (**Figures 3B, C**).

In contrast to the spheroid boundary, the increase of cleaved caspase 8 was detected in the spheroid center, both in controls and in perifosine-treated spheroids (**Figures 4A, B**). This may result from unfavorable conditions in the central zone of the spheroid. This gradient of cleaved caspase 8 distribution was not observed in the spheroids treated with perifosine for 72 h (**Figures 4A, B**). In each time interval tested, the level of cleaved caspase 8 was elevated in the perifosine-positive areas (**Figures 4A–C**). Surprisingly, cleaved caspase 8 was upregulated also in the perifosine-free regions of the spheroid treated with perifosine for 24 h (**Figure 4B**). The possible explanation of this phenomenon is discussed in more detail below (*Discussion section*, paragraph 3).

### Acidosis Decreases Efficacy of Perifosine on HT-29 Spheroids

The spheroids were placed into the control medium (pH 7.4) or the medium supplemented with either NaHCO_3_ (80 mM, pH 8.1) or LA (20 mM, pH 6.6). The pHe on the spheroid surface was determined using cell-impermeable, ratiometric pH indicator SNARF-5F and LSCM analysis (**Supplementary Image 6A**). Even though cultivated in the medium with pH 7.4, the pHe on the surface of the spheroids (up to 15 µm from the boundary) reached the value 6.3. Similar acidification of the spheroid surface to the value of 7.3 and 5.2 was detected for the spheroids exposed to the medium with pH 8.1 and 6.6, respectively (**Supplementary Image 6B**). It documents that the spheroid-forming cells maintain lower pHe on its surface, compared to the pH of the culture media.

**Figure 5:**
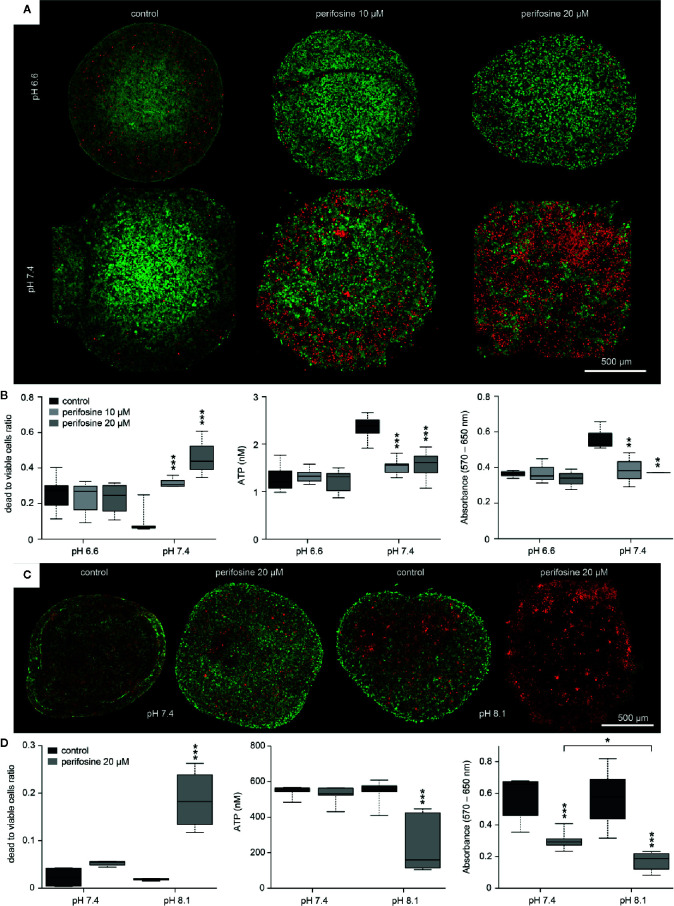
The effect of the tumor-specific environment on cytotoxicity of perifosine to 3D cell cultures. **(A, C)** Images of the control and perifosine-treated (20 µM) spheroids in either acidic, normal, or alkaline media obtained by LSCM after 72 **(A)** and 48 h **(C)**; living cells were dyed using calcein-AM (green) and dead cells using propidium-iodide (red). **(B, D)** Cytotoxicity of perifosine in different tumor-specific environments was calculated as a ratio of dead and living cells after calcein-AM/propidium iodide staining (left), as a change in ATP level (middle) and by MTT assay (right). Results are presented in boxplots showing median, interquartile range, minimum and maximum values; significant difference (*) between controls and perifosine-induced and perifosine-induced samples respectively were evaluated by t-test; *p < 0.05, **p < 0.01, ***p < 0.001.

To analyze the efficacy of perifosine in different intraluminal pH, the spheroids were cultivated in media with pH 6.6 or 7.4 and treated with perifosine for 72 h. Control spheroids were left untreated in both environments. The calcein-AM/propidium iodide staining showed that though acidic conditions induced some cell death even in the controls, perifosine did not cause further increase of the dead/live cell ratio. This suggests that perifosine is not efficient under acidic conditions (**Figures 5A, B**). Results of ATP and MTT assays confirmed that the efficacy of perifosine is reduced in acidic tumor environment (**Figure 5B**).

Compared to pH 7.4, further alkalization of the environment to pH 8.1 for 48 h increased the frequency of dead cells in the perifosine-treated spheroids as documented by the calcein-AM/propidium iodide staining, ATP and MTT assays (**Figures 5C, D**). Spheroids treated in alkalosis for more than 48 h were excluded from analyses due to loss of compactness. We conclude that acidosis significantly decreases cytotoxicity of perifosine to HT-29 cells grown both in the monolayers and in the 3D cultures. Similar results were observed also for the HCT-116 spheroids (**Supplementary Image 7**). This indicates that the modulatory effect of the tumor environment on perifosine efficacy is not cell-line-specific.

**Figure 6:**
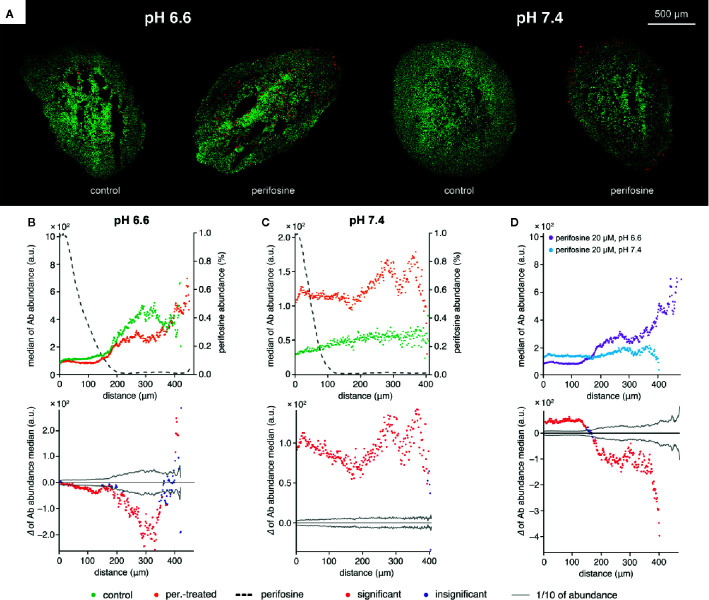
The effect of acidosis on perifosine and cleaved caspase 8 signal abundance and co-localization. **(A)** IHC images showing the signal of cleaved caspase 8 (red) and nuclei (green) for control and perifosine-treated (20 μM) spheroids after 72 h. **(B, C)** Peeling analysis of cleaved caspase 8 and perifosine distributions within the spheroid sections in acidic pH 6.6 **(B)** or normal pH 7.4 **(C)** conditions; distribution of cleaved caspase 8 (median per peel) in the control (•) and perifosine-treated (•) spheroid sections; the perifosine signal was re-scaled using min-max normalization and its distribution is marked by the dashed line (upper part); differences between the median values of cleaved caspase 8 abundance in the control spheroid and perifosine-treated spheroid (lower part). The median value of the control spheroid was subtracted from the median value of the treated spheroid in each peel. **(D)** Comparison of the cleaved caspase 8 distributions (median per peel) in the perifosine-treated spheroids in acidosis (•) and normal (•) media. Differences between the median values of cleaved caspase 8 abundance in the spheroids treated in acidic and normal media (bottom). The median value of the spheroid treated in acidic media was subtracted from the median value of the spheroid treated in normal media. The zero at the X-axis coincides with the outer edge of the spheroid. The significant (•) and insignificant (•) differences of medians for p < 0.05 are distinguished.

To further demonstrate the pH-dependency of the perifosine cytotoxicity, we co-localized perifosine distribution with the cleaved caspase 8-positive areas in the spheroids exposed to normal and acidic conditions (**Figures 6A–C**). We found elevation of the cleaved caspase 8 in the perifosine-treated spheroids only in normal pH, not in the acidosis (**Figures 6A–C**). Then, we compared the level of cleaved caspase 8 in the perifosine-treated spheroids grown in pH 6.6 and 7.4. In acidic conditions, abundance of this apoptotic marker in the perifosine-rich regions was lower, while in the perifosine-free area located 200 µm and more from the spheroid boundary, apoptosis was more pronounced (**Figure 6D**). The same phenomena was observed also in the control spheroids grown in acidosis, where the effect of perifosine was excluded (**Figure 6B**).

**Figure 7:**
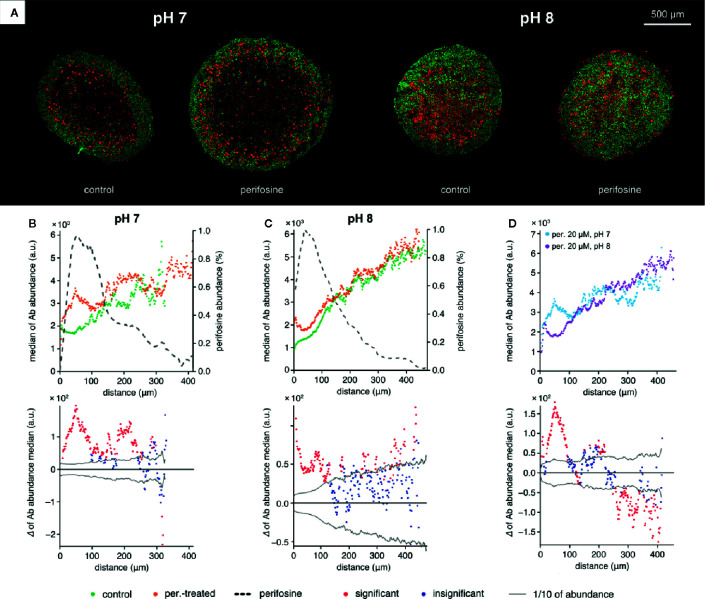
The effect of alkaline conditions on perifosine and cleaved caspase 8 abundance and co-localization in the collagen matrix. **(A)** IHC images showing the signal of cleaved caspase 8 (red) and nuclei (green) for control and perifosine-treated (20 μM) spheroids embedded in collagen matrix after 48 h. **(B, C)** Peeling analysis of cleaved caspase 8 and perifosine distributions within the spheroid sections in normal **(B)** or alkaline **(C)** conditions; distribution of cleaved caspase 8 (median per peel) in the control (•) and perifosine-treated (•) spheroid sections; the perifosine signal was re-scaled using min-max normalization and its distribution is marked by the dashed line (upper part); differences between the median values of cleaved caspase 8 abundance in the control spheroid and perifosine-treated spheroid (lower part). The median value of the control spheroid was subtracted from the median value of the treated spheroid in each peel. **(D)** Comparison of the cleaved caspase 8 distributions (median per peel) in the perifosine-treated spheroids in pH 7 (•) and alkaline pH 8 (•) media; differences between the median values of cleaved caspase 8 abundance in the spheroids treated in normal and alkaline media (bottom). The median value of the spheroid treated in alkaline media was subtracted from the median value of the spheroid treated in normal media. The zero at the X-axis coincides the outer edge of the spheroid. The significant (•) and insignificant (•) differences of medians for p < 0.05 are distinguished.

Finally, we wondered if alkalization of the culture medium can contribute to the perifosine toxicity on spheroids. Therefore, we assesed the distribution of cleaved caspase 8 and perifosine in spheroids exposed to media with pH 7.4 and 8.1 for 48 h. We did not detect any further upregulation of cleaved caspase 8 in the spheroids grown at pH 8.1 compared to pH 7.4 in fluidic conditions (**Supplementary Image 8**). To verify that the cleaved caspase 8-positive cells were not lost during the cultivation in these conditions, the spheroids were embedded and treated in collagen matrix with pH adjusted to 7 and 8 for 48 h. Elevation of cleaved caspase 8 in the perifosine-exposed regions compared to controls was detected in both pH 7 and pH 8 (**Figures 7A–C**). However, elevation of extra-spheroidal pH from 7 to 8 did not further increase the level of cleaved caspase 8; instead, a significant decrease was observed in the perifosine-rich area (**Figures 7B–D**). No difference between perifosine penetration into spheroids exposed to pH 7 and 8 was detected (**Figures 7B, C**). Compared to fluidic conditions (**Supplementary Image 8**), the perifosine signal at the boundary of embedded spheroids was weaker and its penetration was slightly more efficient (**Figure 7B**).

## Discussion

Drug distribution in tumor tissue is important for its clinical efficacy. Only drugs capable of reaching high and homogeneous intratumoral concentrations can significantly reduce tumor growth and angiogenesis (62). Spatial heterogeneity and incomplete drug penetration into tumor tissue facilitate the evolution of multidrug resistance (62, 63). Therefore, uneven intratumoral distribution highlights the need for studies addressing spatial drug distribution within tumors (64). We assessed the perifosine distribution in spheroids derived from colorectal carcinoma cells using MALDI MSI. The perifosine-positive regions were spread within the maximum distance of 200-300 µm from the spheroid boundary with the spheroids ~1,000 µm in diameter, as found in the longest time interval tested (72 h). Within these perifosine-positive regions, the cells were exposed to concentration gradients of this drug. Perifosine concentration variations were determined not only in the direction from the spheroid boundary to its central part, but also within peels. This shows that cells growing at the same distance from the spheroid boundary are exposed to various concentrations of perifosine, as depicted in the graphs of perifosine descriptive statistics (interquartile range, min/max values per peel). This irregular spread of perifosine might result in the development of compartments, in which the cells are exposed to lower dose of perifosine than its effective therapeutic concentration (65). Cells of these compartments might develop resistance to perifosine more frequently, due to stepwise accumulation of mutations (65).

No perifosine was detected deeper than 200 µm from spheroid boundary either in 6 h or in the 24 h time intervals. We investigated whether the perifosine-free regions overlap with the areas of dead cells that might originate from poor supply of oxygen and nutrients in deeper cellular layers (66). Using multi-modal imaging and co-localization workflow, we verified the presence of proliferating cells in the perifosine-free compartments of the perifosine-induced spheroids. Thus, we excluded the possibility that the perifosine-free regions overlap with the areas lacking viable cells. Inability of perifosine to reach tumor cells that are distant from the spheroid boundary suggests mechanism for development of the drug-resistance (44, 64, 67).

As reported previously, maximum inter-vessel distance in human colorectal carcinomas reached 1,000 µm (68). Given the maximum distance of perifosine penetration (200–300 µm) in our experiments, we hypothesize that the perifosine-free regions can be also found in *in vivo* growing tumors. Perifosine penetration into the collagen-embedded spheroids was investigated as well. In contrast to the spheroids exposed to perifosine in fluidic conditions, the maximum perifosine level at the boundary of the collagen embedded spheroids was lower, presumably due to its non-specific binding to the collagen matrix (69). Interestingly, perifosine was detected in the center of the collagen-embedded spheroids, even though at a very low level. Thus, the presence of an extra-tumoral matrix might influence drug distribution within the spheroid.

Determination of drug efficacy in the 3D models might be complicated by the natural presence of apoptotic regions originating from hypoxia, nutrient shortage and/or acidosis. To distinguish the perifosine-induced apoptosis from apoptosis caused by a hostile tumor microenvironment, the precise perifosine distribution in spheroids must be determined. Distributions of the apoptotic regions in the perifosine-treated spheroid and untreated controls need to be compared as well. In the normal pH, the spheroids exhibited regions with both the presence of perifosine and up-regulated markers of apoptosis in each time interval tested. The apoptotic signal was also elevated in the perifosine-free area after 24 h of treatment. This result might be explained by the bystander effect. The bystander effect refers to cell death, altered growth or senescence of cells that had not been directly exposed to ionizing radiation or genotoxic chemicals, such as adriamycin, actinomycin D or mitomycin C, but accepted the medium-soluble or plasma factors (death ligands, inflammatory cytokines, and reactive oxygen species) excreted from the exposed cells. The intercellular signaling between the cells undergoing apoptosis and their neighborhood might reduce the survival rate even in cells, which were not directly exposed (70–75). Thus, perifosine might induce expression of soluble death ligands inducing apoptosis in the perifosine-free areas (71). The level of cleaved caspase 8 detected in spheroid cultivated in the LA-enriched media was lower than in the normal media providing further evidence that acidosis contributes to reduction of the perifosine cytotoxicity.

Ki-67 is strongly associated with cell proliferation and prognosis in CRC patients (76, 77). Therefore, we were interested in the Ki-67 level/distribution in the spheroids treated with perifosine. Surprisingly, increased level of Ki-67 was found in the perifosine-rich areas (in 24 h interval up to 100 µm from the spheroid boundary, in 48 and 72 h intervals 50–150 µm from the spheroid boundary) or localized in the perifosine-free regions (6 and 24 h intervals, 150 µm from spheroid boundary and deeper) in the perifosine-treated sferoids. The mechanism underlying this phenomenon needs to be further investigated. The chemotherapy-induced apoptosis might alleviate solid stress and favor the supply of oxygen and nutrients to the tumor cells resulting in the proliferation of drug-resistant cells or cells localized in the drug-free layers (78, 79). Indeed, the perifosine-treated spheroids are less compact than the controls and their periphery is composed of loosely attached cells. Therefore, perifosine might improve the supply of nutrients to the spheroid cells (**Supplementary Image 9**). The chemotherapy-induced cell proliferation has been observed previously and might contribute to chemotherapy failure (79, 80). However, perifosine downregulates Ki-67 during 48 and 72 h, and this effect is spatially restricted to the most peripheral cell layers 0–50 µm from the spheroid boundary. This indicates that perifosine should be administered for an extended period of time to decrease cell proliferation marked by Ki-67.

Perifosine penetration into spheroids is limited, as documented by spheroids derived from HT-29 cells. An inverse relationship between drug penetration and its binding to cellular components was reported (81). Perifosine accumulates in the cell membranes, thus, it belongs to the group of drugs with strong binding to cellular macromolecules, similar to doxorubicin, daunomycin, actinomycin D, and others (64, 82). Therefore, the slow delivery of perifosine to the inner cells of spheroids might be attributable to its efficient binding to cells in the outer layers (83).

Analysis of perifosine distribution, cell proliferation, and apoptosis reveals great heterogeneity in perifosine uptake and phenotypic response even within the same layer. This is exemplified in **Figures 3** and **4**, showing both cleaved caspase 8 and Ki-67 elevated at the distance of 50 µm from the spheroid boundary in the spheroids treated with perifosine for 48 h. The spheroids were derived from cell lines comprising of mixtures of phenotypically distinct subpopulations (84–87). These cell subpopulations might respond variously to drug application, for example, they might uptake perifosine with different efficacy. Also, even the cells with similar perifosine accumulation might respond dissimilarly, inducing either apoptosis or drug-resistant phenotype (84, 85, 88).

Drug efficacy may be seriously hampered by the tumor microenvironment. Acidosis, hypoxia, and nutrient shortage are common factors affecting chemotherapy effectiveness (30, 31, 89, 90). We have also previously reported increased cytotoxicity of the disulfiram/copper complex in acidosis and tetrathiomolybdate in low-glucose conditions (54, 55). It is well established that in tumors, acidosis can result from poor perfusion and accumulation of lactate in deep tumor layers (30, 91). Besides, as often neglected in CRC, colorectal tumors can be localized in colon regions with various intraluminal pH values (92). Using multiple assays, we proved that pH less than 6.7 significantly interferes with perifosine efficacy, both in cells in monolayers as well as spheroids. Elevation of extra-tumoral pH potentiated cytotoxicity of perifosine. However, the levels of cleaved caspase 8 in the spheroids exposed to perifosine in pH 7.4 and 8.1 in fluidic conditions or 7 and 8 in the collagen matrix, respectively, were comparable. The interpretation of this observation requires further experiments, but presumably the caspase 8-independent apoptotic and pro-death signaling pathway might be activated by perifosine in the spheroids exposed to highly alkaline conditions. In cellular monolayers, acidosis also interfered with intracellular accumulation of perifosine, both in normoxia and hypoxia. The mechanism behind the pH-dependent perifosine accumulation remains to be elucidated. We hypothesize that perifosine can be internalized by the phospholipid translocase activity or raft- and dynamin-mediated endocytosis (9, 93). Since especially the translocase activity strongly depends on ATP and the ATP level is decreased in cells exposed to acidosis, the absence of the translocase activity in the ATP-depleted acidic compartments might result in the lower accumulation of perifosine. However, the lack of perifosine accumulation in cells exposed to acidic pH cannot easily explain the loss of its cytotoxicity; the active form of the major perifosine target, the Akt kinase, is decreased to a similar extent in normal and acidic pH (**Supplementary Image 4**).

The untreated controls in pH 6.6 differed from those at pH 7.3, 7.4 and 7.9 in MTT and ATP level. It can be explained by the lower proliferation rate of cells cultivated in acidosis compared to those exposed to more alkaline conditions. The slower proliferation of these cells can result from the acidosis-induced “anti-Warburg” metabolic effects, starvation response and mTORC1 inhibition (94, 95).

The Akt kinase exerts its anti-apoptotic effect through multiple mechanisms, such as stimulation of glycolysis and glucose metabolism, regulation of the Bcl-2 proteins, and hexokinase activity (96–99). We hypothesize that the metabolism of cells exposed to acidosis might not be sensitive to the Akt kinase inhibition, as acidosis directs energy generation from the Akt-dependent glucose metabolism and glycolysis toward aerobic respiration (30, 94, 100, 101) and glutamine utilization (102). Likewise, during the preparation of this manuscript, Barnes et al. reported that lactic acidosis interferes with cytotoxicity of another Akt inhibitor, uprosertib, in colon cancer cells (30). They suggest the ability to utilize lactate as a carbon source, decoupled glycolysis from the Citrate cycle, and enhanced oxidative metabolism as possible mechanisms of resistance of CRC cells to Akt inhibition in acidosis (30). Furthermore, the induction of chronic autophagy belongs to the survival adaptation of cells exposed to acidic conditions (103). Since protective autophagy is curtailed by the Akt signaling, cells in acidosis might benefit from Akt inhibition (104). Apart from this, in normal pH, phosphorylated Akt activates mTORC1, the master regulator of cellular proliferation and NFκB, whose abberant activation leads to tumorigenic potential of cancer cells (105, 106). However, acidosis reduces both mTORC1 activity and the efficacy of its inhibitor, rapamycin (107). Moreover, in low pH, NFκB regulation is diverted from Akt kinase to alternative ASIC1-ROS-ERK-IκBα axis (108). Thus, decreased efficacy of Akt inhibitors in low pH might be explained by pH-dependent regulation of signaling pathways linked to this kinase. Limited efficacy of the Akt inhibitors to cells grown in acidosis was shown by our group also for the other Akt inhibitor, MK-2206 (**Supplementary Image 10**). Therefore, the effect of the tumor-specific microenvironment should be considered in design and preclinical studies of Akt inhibitors in CRC therapy.

## Conclusions

Our experiments confirmed the critical role of perifosine spatial distribution for the evaluation of its efficacy in spheroids. Distribution of perifosine inside the spheroids was found to be very heterogeneous, and the depth of penetration was time-dependent. Changes in the Ki-67 and cleaved caspase 8 distribution resulting from perifosine administration were interpreted in the context of 3D objects. We document that the drug-induced pro-apoptotic effect might be associated with increased proliferation of the drug-resistant cells as a result of changes in cellular density and presumably, nutrient supplementation. Moreover, the suppressive effect of acidosis on cytotoxicity of perifosine to cancer cells growing in monolayers and spheroids and its reversibility by alkalization was highlighted. As lactic acidosis interferes with cytotoxicity of the other Akt kinase inhibitors, external tumor pH should be considered in further design of the Akt-targeted treatment strategy.

## Data Availability Statement

The raw data supporting the conclusions of this article will be made available by the authors, without undue reservation.

## Author Contributions

JN, JP, and MK contributed to the conception of the study. BP, MM, PBr, AP, and JN performed the experiments. BP and JN wrote the manuscript. KŠ, JM, and MK developed the fiducial-marker-based co-registration workflow and performed the image analysis. TN was responsible for the statistical evaluation. AP helped significantly with graphic processing. PBe helped formulate clear conclusions with constructive discussion and JŠ contributed to the final coherence of text and by language skills. All authors contributed to the article and approved the submitted version.

## Funding

This work was supported by the Grant Agency of Masaryk University (MUNI/G/0974/2016 and MUNI/A/1127/2019) and the Ministry of Education, Youth and Sports of the Czech Republic (MEYS CR) under the projects CEITEC 2020 (LQ1601), TRANS-MED (LQ1605), the core facility CELLIM of CEITEC supported by the Czech-BioImaging large RI project (LM2018129 funded by MEYS CR) and ERDF (No. CZ.02.1.01/0.0/0.0/16_013/0001775).

## Conflict of Interest

The authors declare that the research was conducted in the absence of any commercial or financial relationships that could be construed as a potential conflict of interest.

## Abbreviations

ATP, adenosine triphosphate; calcein-AM, calcein-acetoxymethyl; CRC, colorectal cancer; DHB, 2,5-dihydroxybenzoic acid; DMEM, Dulbecco’s Modified Eagle’s Medium; DMSO, dimethyl sulfoxide; FBS, fetal bovine serum; IHC, immunohistochemistry; IQR, interquartile range; ITO, indium tin oxide; LA, lactic acid; LSCM, laser scanning confocal microscopy; MALDI TOF MS, matrix-assisted laser desorption/ionization time-of-flight mass spectrometry imaging; MSI, mass spectrometry imaging; MTT, 3-(4,5-dimethylthiazol-2-Yl)-2,5-diphenyltetrazolium bromide; NaL, sodium lactate; per.-treated, perifosine-treated; PBS, phosphate-buffered saline; pHe, extracellular pH; PI3K, phosphoinositide 3-kinase; pKa, dissociation constant; RPMI, Roswell Park Memorial Institute; SD, standard deviation.
