# Supplementary material for: Lactic Acidosis Interferes With Toxicity of Perifosine to Colorectal Cancer Spheroids: Multimodal Imaging Analysis
Source: Front Oncol. 2020 Dec 4;10:581365. doi: 10.3389/fonc.2020.581365 (PMC7746961; doi:10.3389/fonc.2020.581365)
Supplement: Supplementary file 1 [file DataSheet_1.pdf]

Methodology: MALDI TOF MS of 2D and 3D cell cultures

Matrix-Assisted Laser Desorption/Ionization Time-of-Flight Mass Spectrometry (MALDI TOF MS) of 2D and 3D cell cultures

Samples were analyzed by MALDI TOF MS (Autoflex Speed; Bruker, Massachusetts, USA) in positive reflector mode as described recently (51). Very briefly, the m/z range was set to 3401000 to detect primarily perifosine (m/z 462.45), fiducial markers (whole m/z range) and phospholipids (m/z 500-1000). The high 2, 5-dihydroxybenzoic acid (DHB) matrix signals were deflected up to m/z 320. The imaging analyses were proceeded by FlexImaging 5.0 software (Bruker, Massachusetts, USA).

Spatial Distribution, Co-localization and Quantification of MALDI MSI and IHC signal in spheroid sections

The protocol of spheroid preparation for MALDI MSI and LSCM analysis was described in detail recently (51). Briefly, the spheroids were transferred to plastic cryomolds (Tissue-Tek® Cryomold®; Sakura Finetek, California, USA) with warm gelatine solution (180 mg/mL in 1x PBS, 40 °C) and frozen in -80 °C. The gelatine blocks were cut in a cryostat microtome CM1850 (Leica Microsystems, Wetzlar, Germany) into 12-µm thick equatorial cross sections and these were collected on the ITO conductive slides (Delta Technologies, Limited; Colorado, USA). Each analyzed spheroid was labeled by three fiducials with special composition suitable for the IHC-compatible MALDI MSI analysis (51). The ITO slides were covered by DHB matrix (98%; Sigma Aldrich, Missouri, USA) and the samples were analyzed by MALDI MSI the same day. Next day, matrix was removed and the fluorescent IHC protocol was performed. The sections were incubated with primary antibodies specific for the Ki-67 protein (ab16667, Abcam, Cambridge, UK) and cleaved caspase 8 (CST9496s, Cell Signalling; Massachusetts, USA). Next day, secondary fluorescently labelled antibody (Alexa 546; Invitrogen, California, USA) and TO-PRO™-3 Iodide (T3605, Invitrogen, California, USA) were applied for 1-2 hours in the dark. Finally, mounting medium (Dako fluorescent mounting medium; Agilent, California, USA) with coverslip (Menzel Gläser; Thermo Scientific, Massachusetts, USA) was applied on the sections. Samples were kept in dark and cold conditions until the LSCM (TCS SP8; Leica Microsystems, Wetzlar, Germany) analysis. To obtain complete fluorescence signals from all optical layers, Z-stacks with a vertical spacing of 4 µm were acquired. To eliminate the fluorescence signal resulting from the autofluorescence and nonspecific binding of a secondary antibody, the fluorescence signal obtained from the spheroid section incubated with only the secondary antibody was subtracted from the signal obtained from sections stained by the primary-secondary antibody complex. Final images were obtained by LAS X software (Leica Microsystems, Wetzlar, Germany). Then, the MS and IHC images were registered using a fiducial-marker-based approach (50, 51).
