# Supplementary material for: Lactic Acidosis Interferes With Toxicity of Perifosine to Colorectal Cancer Spheroids: Multimodal Imaging Analysis
Source: Front Oncol. 2020 Dec 4;10:581365. doi: 10.3389/fonc.2020.581365 (PMC7746961; doi:10.3389/fonc.2020.581365)
Supplement: Supplementary file 2 [file Image_1.pdf]

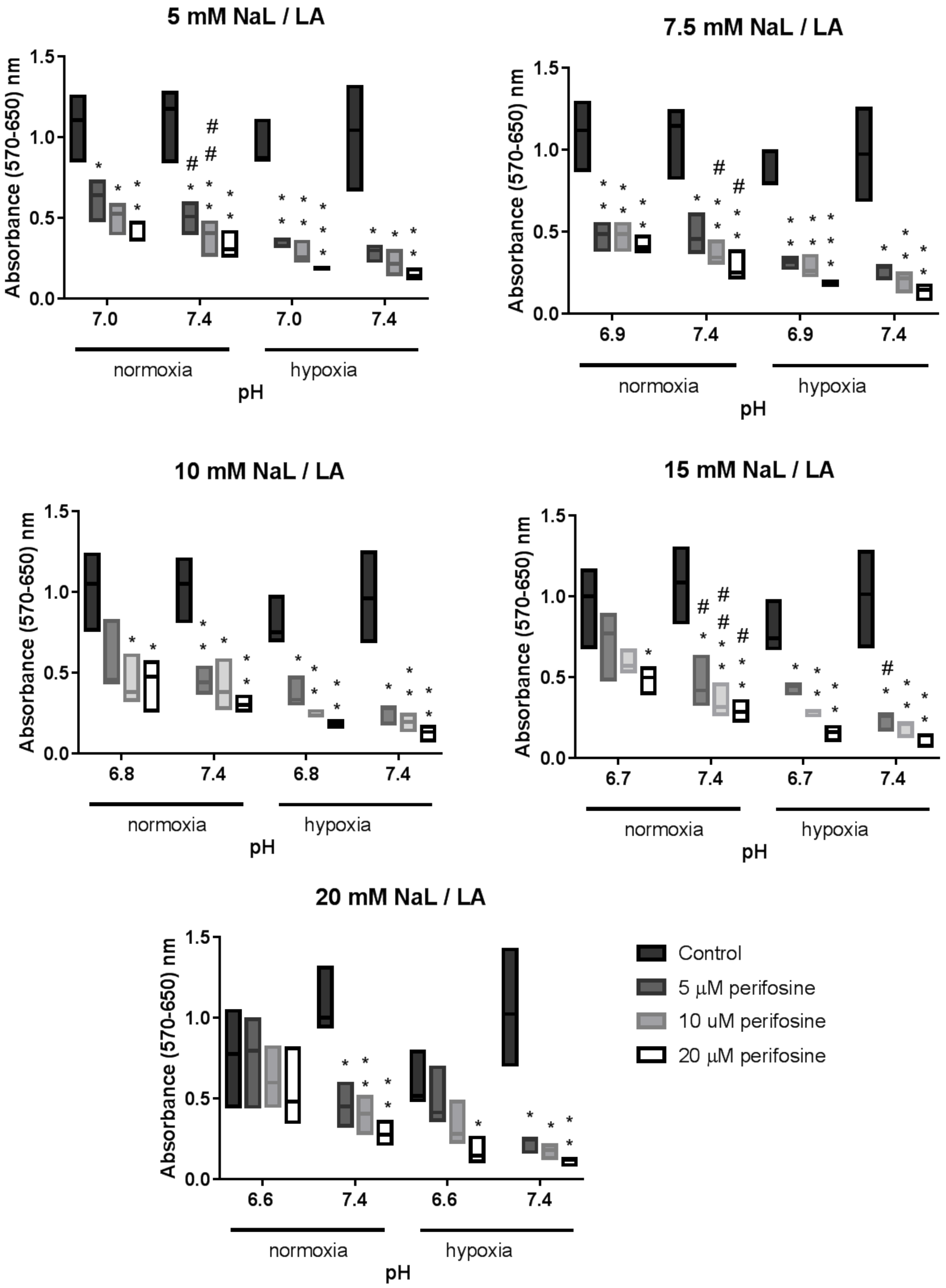

Supplementary Figure 1A: pH-dependent perifosine efficacy. HT-29 cells (2x10^5 cells/mL) were seeded in cultivation medium enriched by either 5, 7.5, 10, 15, and 20 mM lactic acid or sodium lactate to create a gradient of pH from acidosis (pH 6.6) to normal conditions (pH 7.4). The cells were treated by perifosine for 48 hours in normoxia or hypoxia. After that, the MTT assay was performed. The values of pH are marked on the x axis. Statistical significance was evaluated by t-test, */# $p < 0.05$, **/## $p < 0.01$, *** $p < 0.001$. Statistical difference between control and perifosine-treated cells (*), statistical difference between the cells treated by perifosine in pH 7.4 and pH more acidic than 7.4 (#). Three independent biological replicates were made.

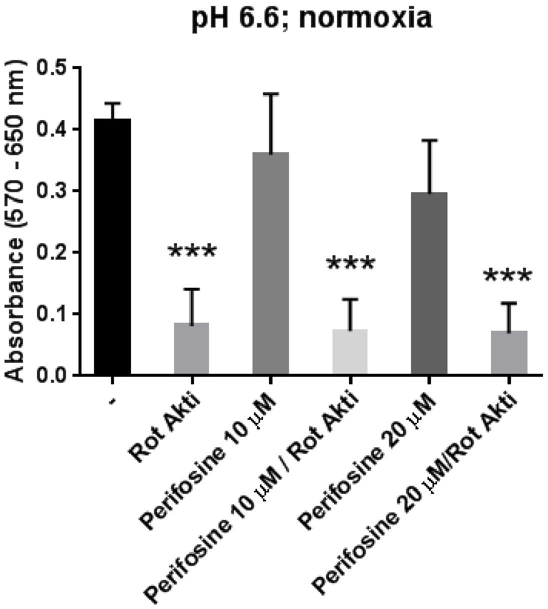

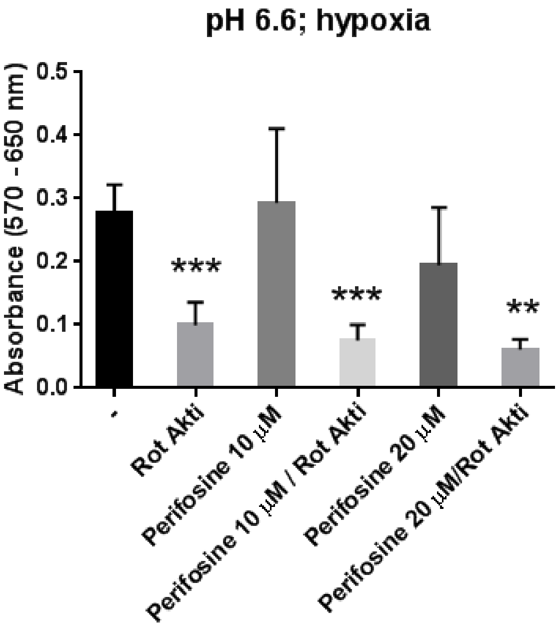

Supplementary Figure 1B: Metabolic activity of HT-29 cells exposed to inhibitor of glucose uptake (Akti1/2) and OXPHOS (rotenone). HT-29 cells were cultivated in pH 6.6 in either normoxia or hypoxia for 72 h. Then perifosine alone or in combination with both Akti1/2 (Akti, 20 μM) and rotenone (Rot, 200 nM) was added for another 72 h. The viability was evaluated by MTT test. Statistical significance was evaluated by t-test, * $p < 0.05$, ** $p < 0.01$, *** $p < 0.001$ indicates difference between untreated controls and controls with Akti1/2 and rotenone or perifosine alone and perifosine in combination with Akti1/2 and rotenone. Three independent biological replicates were made.
