# Supplementary material for: Lactic Acidosis Interferes With Toxicity of Perifosine to Colorectal Cancer Spheroids: Multimodal Imaging Analysis
Source: Front Oncol. 2020 Dec 4;10:581365. doi: 10.3389/fonc.2020.581365 (PMC7746961; doi:10.3389/fonc.2020.581365)
Supplement: Supplementary file 3 [file Image_2.pdf]

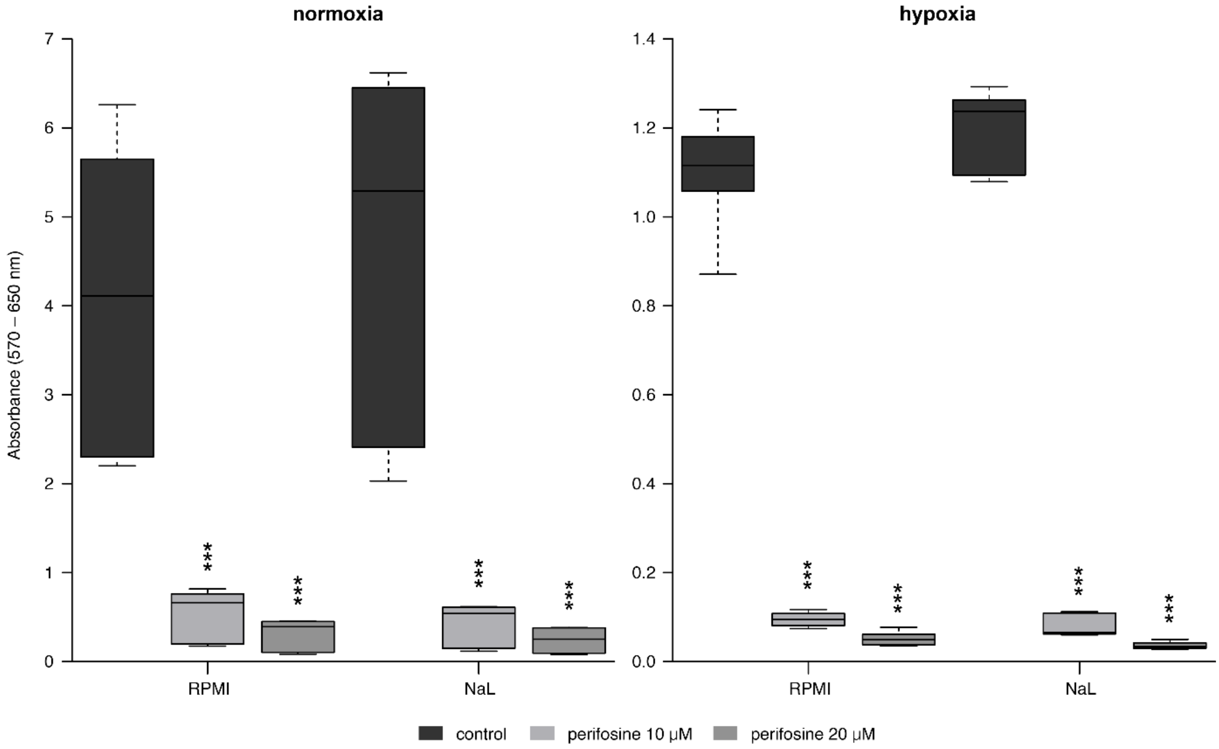

Supplementary Figure 2: Cytotoxicity of perifosine in RPMI media compared to media with sodium lactate. HT-29 monolayers were cultivated in RPMI medium alone or supplemented with sodium lactate (NaL) and treated with perifosine for 72 h in normoxia or hypoxia. After that, the cell viability was investigated by MTT assay. Results are presented in boxplots showing median, interquartile range, minimum and maximum values; significant difference (*) between controls and perifosine-induced samples was evaluated by t-test, *** $p < 0.001$.
