# Supplementary material for: Lactic Acidosis Interferes With Toxicity of Perifosine to Colorectal Cancer Spheroids: Multimodal Imaging Analysis
Source: Front Oncol. 2020 Dec 4;10:581365. doi: 10.3389/fonc.2020.581365 (PMC7746961; doi:10.3389/fonc.2020.581365)
Supplement: Supplementary file 4 [file Image_3.pdf]

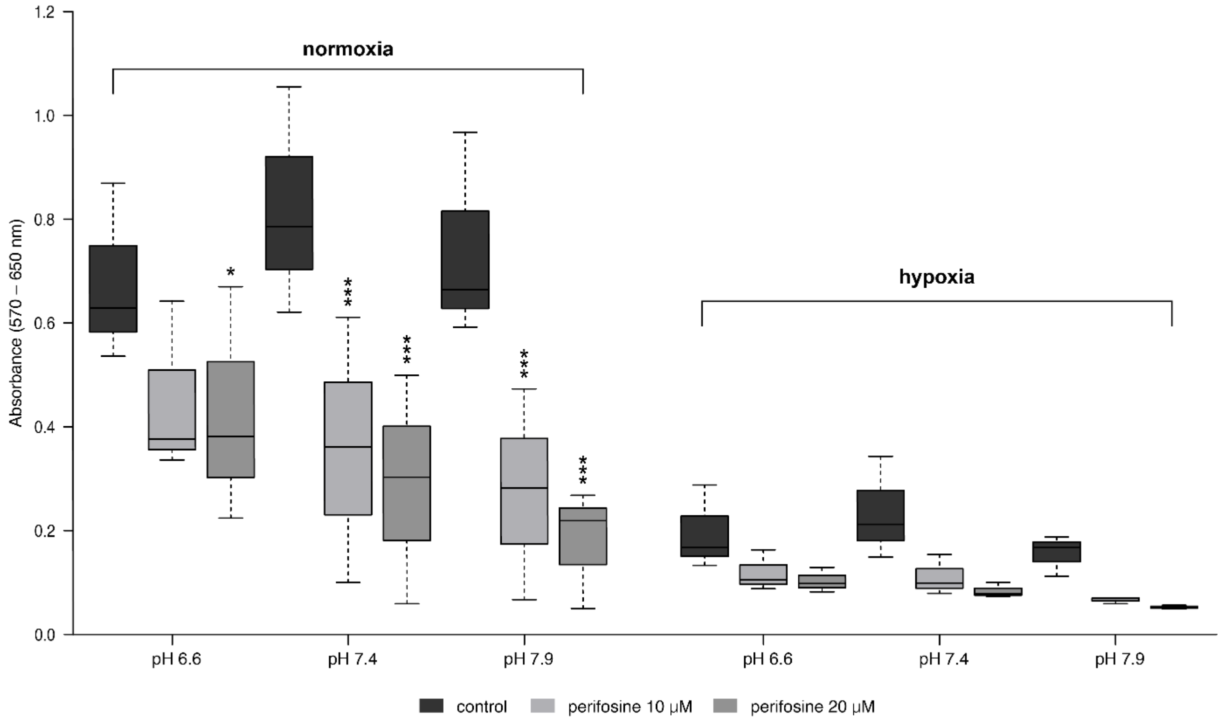

Supplementary Figure 3: pH-dependent cytotoxicity of perifosine to monolayers of HCT116 cells. The cells were exposed to different pH in normoxia and hypoxia. After 72 h, perifosine was added for another 24 h and then, its cytotoxicity was determined by MTT assay. Results are presented in boxplots showing median, interquartile range, minimum and maximum values; significant difference (*) between controls and perifosine-induced samples was evaluated by t-test, * $p < 0.05$, *** $p < 0.001$.
