# Supplementary material for: Lactic Acidosis Interferes With Toxicity of Perifosine to Colorectal Cancer Spheroids: Multimodal Imaging Analysis
Source: Front Oncol. 2020 Dec 4;10:581365. doi: 10.3389/fonc.2020.581365 (PMC7746961; doi:10.3389/fonc.2020.581365)
Supplement: Supplementary file 5 [file Image_4.pdf]

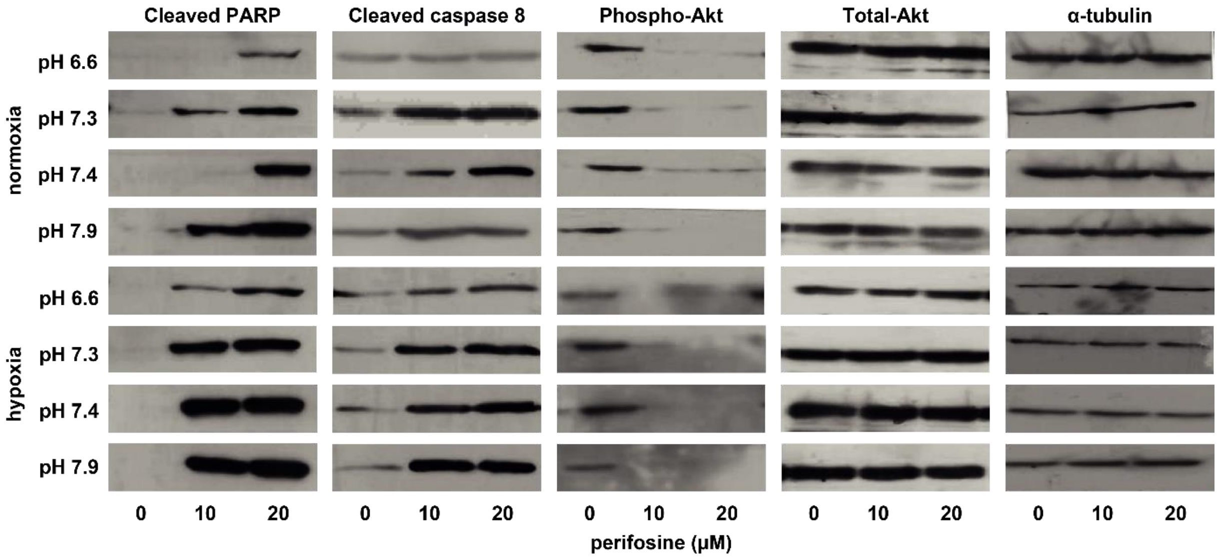

Supplementary Figure 4: Immunoblottings of the cleaved PARP, cleaved caspase 8, phospho-Akt and total Akt intracellular level. HT-29 monolayers were cultivated in different pH either in normoxia or hypoxia. After 72 h, perifosine was added for another 24 h. Then, the samples were subjected to immunoblotting analysis.
