# Supplementary material for: Lactic Acidosis Interferes With Toxicity of Perifosine to Colorectal Cancer Spheroids: Multimodal Imaging Analysis
Source: Front Oncol. 2020 Dec 4;10:581365. doi: 10.3389/fonc.2020.581365 (PMC7746961; doi:10.3389/fonc.2020.581365)
Supplement: Supplementary file 6 [file Image_5.pdf]

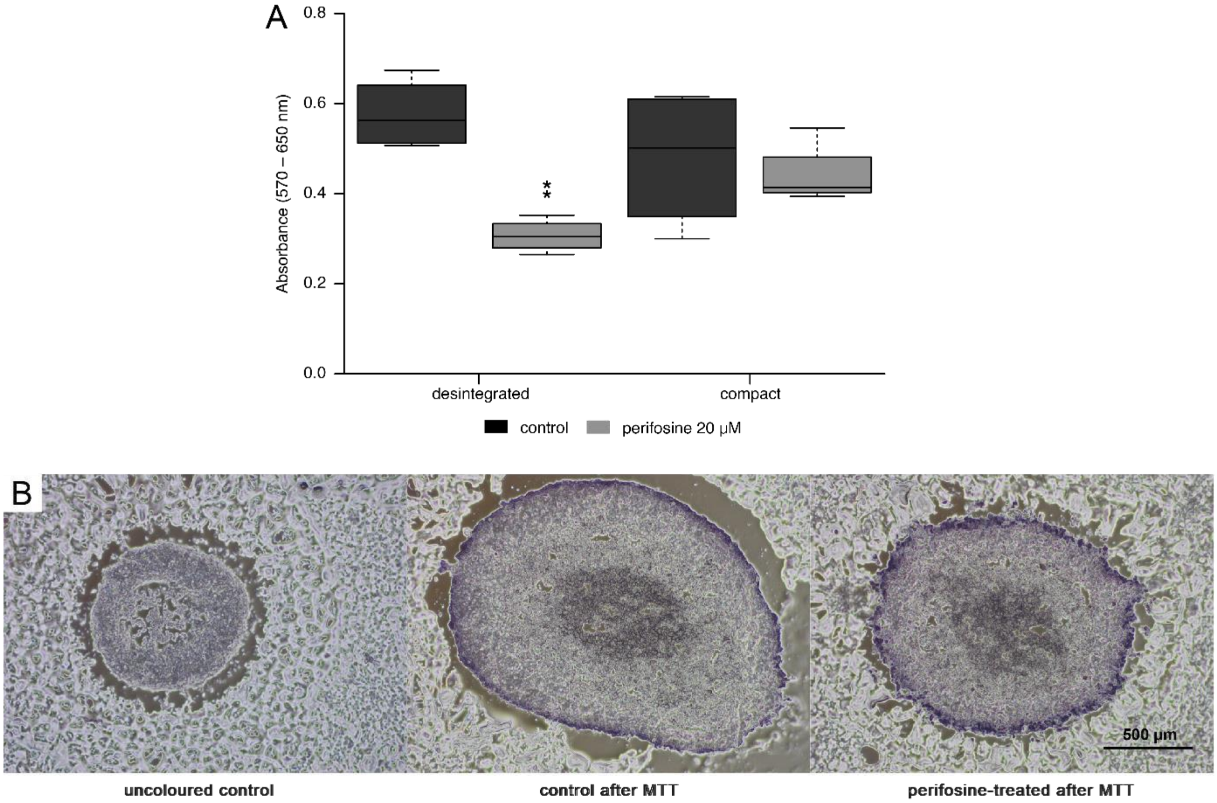

Supplementary Figure 5: Optimization of the MTT assay in 3D tumor models. (A) HT-29 spheroids were induced by perifosine for 72 h, then were either dissociated using trypsin or preserved without trypsin and exposed to MTT for 2.5 h. Results are presented in boxplots showing median, interquartile range, minimum and maximum values; significant difference (*) between controls and perifosine-induced samples was evaluated by t-test, ** $p < 0.01$. (B) The spheroids without trypsin dissociation were placed into gelatine, frozen and cut. The MTT distribution within the spheroid section was analyzed by the brightfield microscopy.
