# Supplementary material for: Lactic Acidosis Interferes With Toxicity of Perifosine to Colorectal Cancer Spheroids: Multimodal Imaging Analysis
Source: Front Oncol. 2020 Dec 4;10:581365. doi: 10.3389/fonc.2020.581365 (PMC7746961; doi:10.3389/fonc.2020.581365)
Supplement: Supplementary file 7 [file Image_6.pdf]

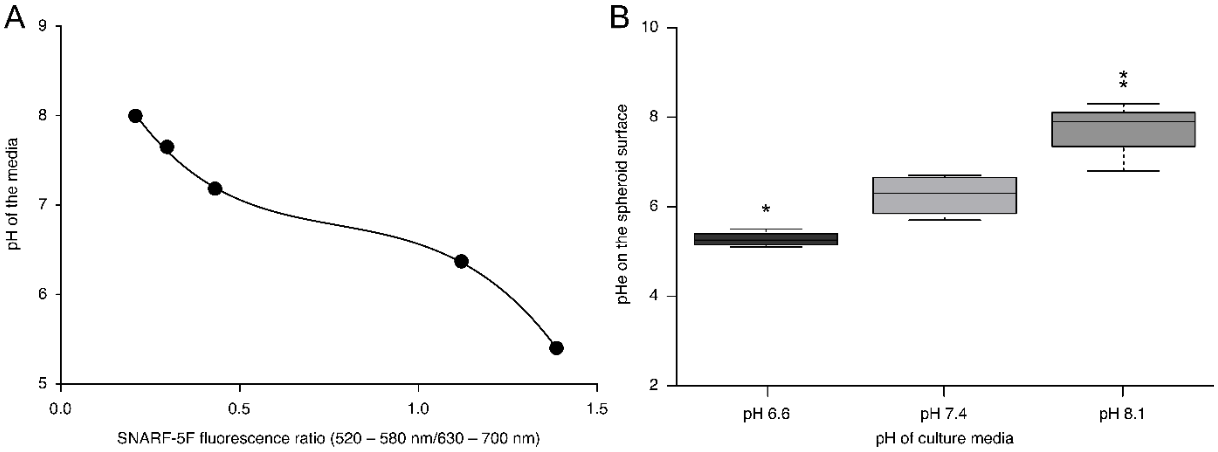

Supplementary Figure 6: Measurement of the pHe on the spheroid surface. (A) The calibration curve for cell-impermeant SNARF-5F probe was established. Horizontal axis shows ratios of SNARF-5F fluorescence, vertical axis represents pH of cultivation media. (B) HT-29 spheroids were cultivated in media with pH 6.6; 7.4; 8.1, as determined by PreSens technology. The pHe on the spheroid surface (in depth 15 µm from the spheroid boundary) was evaluated by SNARF-5F labeling and LSCM. Results in B are presented in boxplots showing median, interquartile range, minimum and maximum values; significant difference (*) between spheroids exposed to pH 7.4 and those cultured in pH 6.6 and 8.1 was evaluated by t-test, * $p < 0.05$, ** $p < 0.01$.
