# Supplementary material for: Lactic Acidosis Interferes With Toxicity of Perifosine to Colorectal Cancer Spheroids: Multimodal Imaging Analysis
Source: Front Oncol. 2020 Dec 4;10:581365. doi: 10.3389/fonc.2020.581365 (PMC7746961; doi:10.3389/fonc.2020.581365)
Supplement: Supplementary file 8 [file Image_7.pdf]

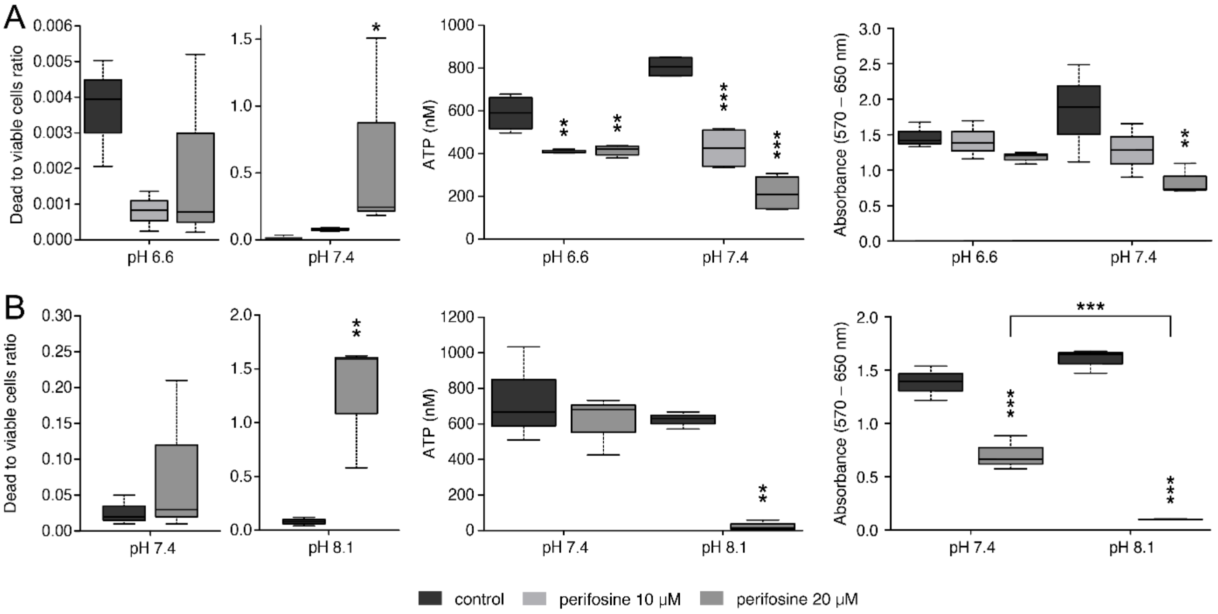

Supplementary Figure 7: pH-dependent cytotoxicity of perifosine to the spheroids derived from HCT-116 cells. The spheroids were treated by perifosine in normal or acidic conditions for 72 hours (A), or in the alkalosis for 48 hours (B). The cytotoxicity of perifosine was investigated as a ratio of dead and viable cells after calcein-AM/propidium-iodide staining (left), as a change in ATP level (middle) and by MTT assay (right). Results are presented in boxplots showing median, interquartile range, minimum and maximum values; significant difference (*) between controls and perifosine-induced samples (or perifosine-induced samples in different pH conditions, respectively) were evaluated by t-test, * $p < 0.05$, ** $p < 0.01$, *** $p < 0.001$.
