# Supplementary material for: Lactic Acidosis Interferes With Toxicity of Perifosine to Colorectal Cancer Spheroids: Multimodal Imaging Analysis
Source: Front Oncol. 2020 Dec 4;10:581365. doi: 10.3389/fonc.2020.581365 (PMC7746961; doi:10.3389/fonc.2020.581365)
Supplement: Supplementary file 9 [file Image_8.pdf]

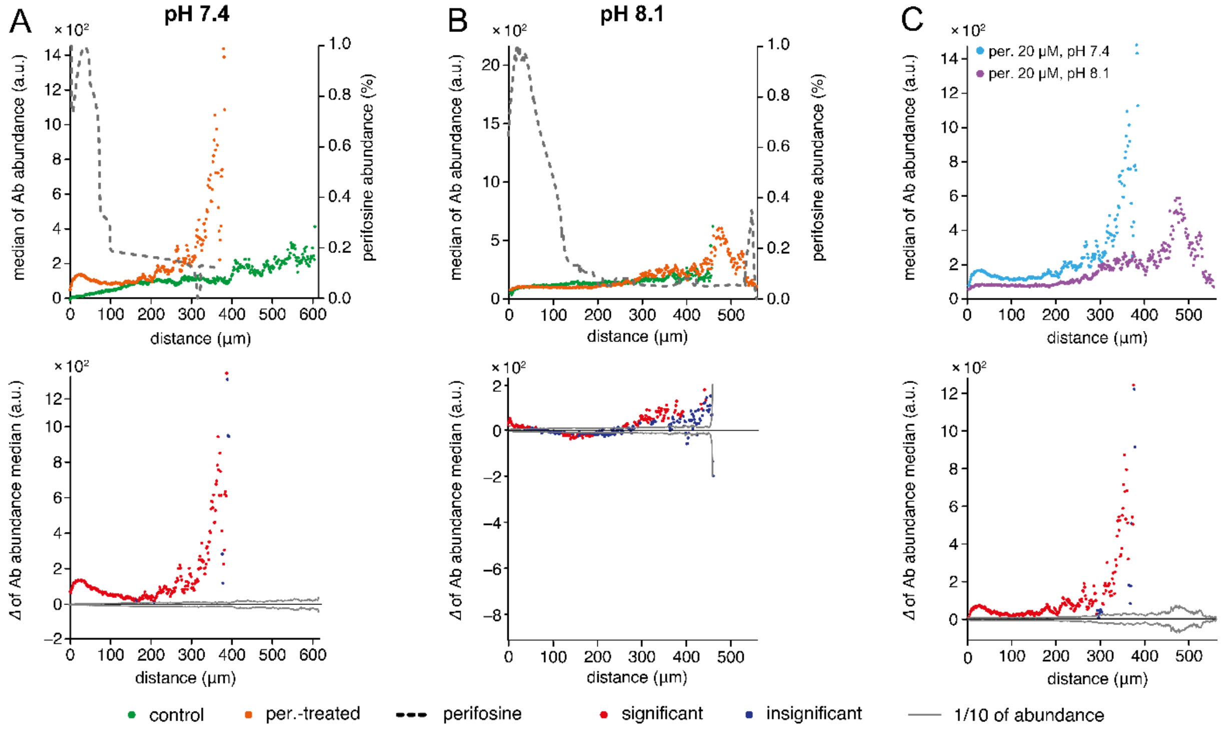


**Supplementary Figure 8: The effect of alkaline conditions on perifosine and cleaved caspase 8 abundance and co-localization in the floating medium.** (A+B) Peeling analysis of cleaved caspase 8 and perifosine distributions within the spheroid sections in normal (A) or alkaline (B) conditions; distribution of cleaved caspase 8 (median per peel) in control (●) and perifosine-treated (●) spheroid sections; the perifosine signal was re-scaled using min-max normalization and its distribution is marked by the dashed line (upper part); differences between the median values of cleaved caspase 8 abundance in the control spheroid and perifosine-treated spheroid (lower part). The median value of the control spheroid was subtracted from the median value of the treated spheroid in each peel. (C) Comparison of the cleaved caspase 8 distributions (median per peel) in the perifosine-treated spheroids in normal pH 7.4 (●) and alkaline pH 8.1 (●) media (top); differences between the median values of antibody abundance in the spheroids treated in normal and alkaline media (bottom). The median value of the spheroid treated in alkaline media was subtracted from the median value of the spheroid treated in normal media. The zero at the X-axis coincides the outer edge of the spheroid. The significant (●) and insignificant (●) differences of medians for $p < 0.05$ are distinguished.
