# Supplementary material for: Lactic Acidosis Interferes With Toxicity of Perifosine to Colorectal Cancer Spheroids: Multimodal Imaging Analysis
Source: Front Oncol. 2020 Dec 4;10:581365. doi: 10.3389/fonc.2020.581365 (PMC7746961; doi:10.3389/fonc.2020.581365)
Supplement: Supplementary file 10 [file Image_9.pdf]

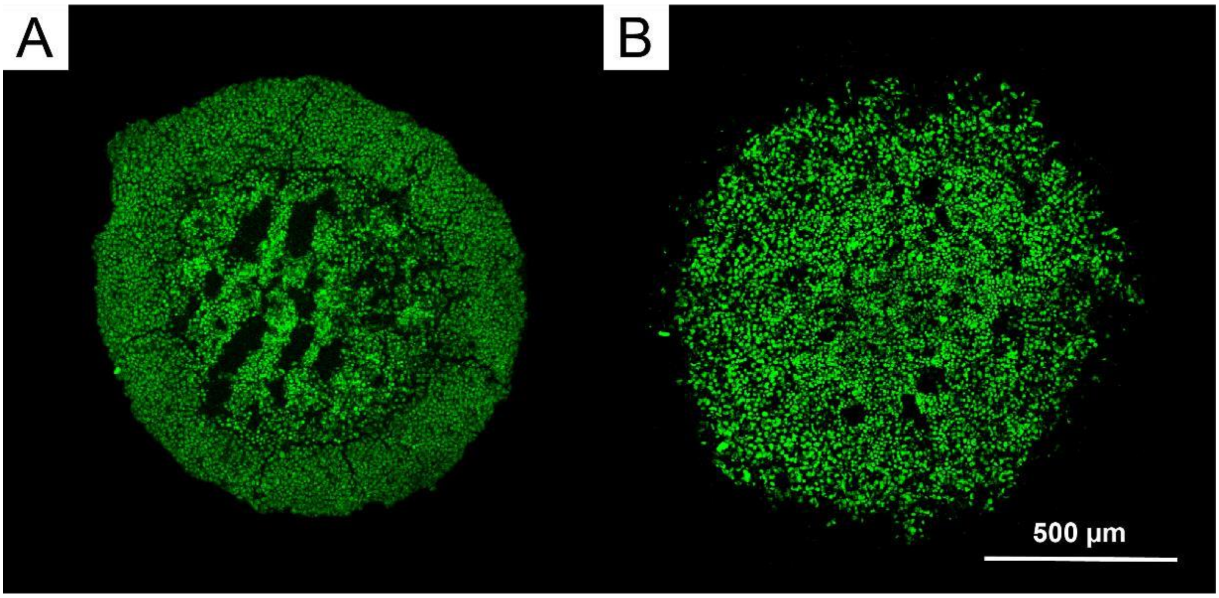

Supplementary Figure 9: Perifosine-induced changes in spheroid compactness. Spheroids derived from HT-29 cells were left untreated (A) or were treated by perifosine for 72 hours (B) Then, the spheroids were frozen, cut and the cell nuclei were stained by TO-PRO (green).
