# Supplementary material for: Lactic Acidosis Interferes With Toxicity of Perifosine to Colorectal Cancer Spheroids: Multimodal Imaging Analysis
Source: Front Oncol. 2020 Dec 4;10:581365. doi: 10.3389/fonc.2020.581365 (PMC7746961; doi:10.3389/fonc.2020.581365)
Supplement: Supplementary file 11 [file Image_10.pdf]

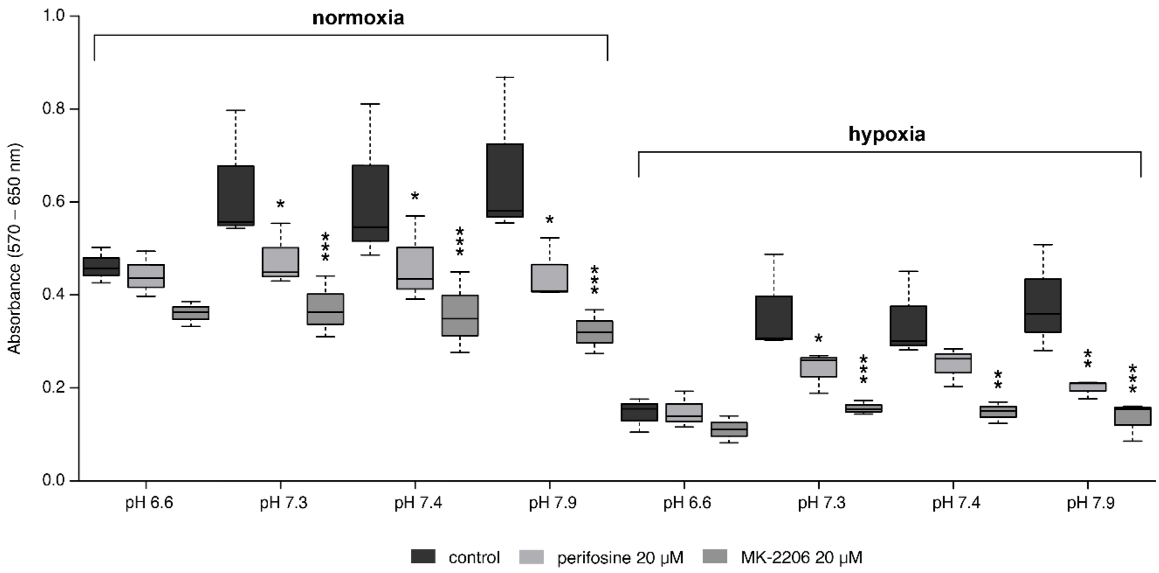

Supplementary Figure 10: pH-dependent efficacy of MK-2206. Monolayers of HT-29 cells were exposed to the different pH conditions in normoxia and hypoxia. After 72 h, the cells were induced with 20 µM MK-2206 or with perifosine for 24 h and the viability was assessed by MTT test. Results are presented in boxplots showing median, interquartile range, minimum and maximum values; significant difference (*) between controls and perifosine- or MK-2206-induced samples was evaluated by t-test, * $p < 0.05$, ** $p < 0.01$, *** $p < 0.001$.
