# Supplementary material for: Lactic Acidosis Interferes With Toxicity of Perifosine to Colorectal Cancer Spheroids: Multimodal Imaging Analysis
Source: Front Oncol. 2020 Dec 4;10:581365. doi: 10.3389/fonc.2020.581365 (PMC7746961; doi:10.3389/fonc.2020.581365)
Supplement: Supplementary file 12 [file Table_1.pdf]

Supplementary Table 1: Cell cycle analysis by flow cytometry. HT-29 monolayers were exposed to different pH either in normoxia or hypoxia for 72 h. Then, perifosine was added for another 24 h. After that, to resolve the subG0/G1 phase of the cell cycle, the extraction of low-molecular-weight DNA was performed using citrate buffer. The percentage of the cells in different phases of cell cycle was analyzed by flow cytometry. Populations in each phase of the cell cycle in each environment were compared to the corresponding value in pH 6.6. Statistical significance was evaluated by t-test, ** $p < 0.01$, *** $p < 0.001$.

**subG0/G1**	pH	20 µM perifosine	Median of % cell population (min-max)		pH	20 µM perifosine	Median of % cell population (min-max)
normoxia	6.6	-	3.7 (2.3-4.8)	hypoxia	6.6	-	7.1 (4.9-11.5)
		+	12.9 (12.4-22.4)			+	12.8 (11.7-16.8)
	7.3	-	2.0 (1.3-4.7)		7.3	-	3.3 (2.8-3.7)
		+	**50.8 (32.1-54.7) *** **			+	**46.8 (26.0-58.2) *** **
	7.4	-	2.1 (1.5-3.3)		7.4	-	8.4 (5.4-10.8)
		+	**47.4 (30.3-54.4) *** **			+	**46.4 (25.5-49.6) *** **
	7.9	-	1.8 (1.3-3.0)		7.9	-	3.4 (3.1-4.9)
		+	**47.5 (39.1-48.0) *** **			+	**42.1 (38.4-52.7) *** **

**G0/G1**	pH	20 µM perifosine	Median of % cell population (min-max)		pH	20 µM perifosine	Median of % cell population (min-max)
normoxia	6.6	-	64.6 (52.1-66.4)	hypoxia	6.6	-	45.7 (23.8-56.7)
		+	38.6 (34.2-43.0)			+	52.3 (41.0-62.6)
	7.3	-	69.2 (58.0-70.6)		7.3	-	39.4 (35.2-59.7)
		+	27.1 (22.4-27.3)			+	31.2 (28.0-53.3)
	7.4	-	71.5 (59.3-72.6)		7.4	-	48.9 (37.9-70.6)
		+	25.3 (20.0-26.7)			+	39.2 (24.7-52.8)
	7.9	-	71.0 (54.8-72.7)		7.9	-	59.7 (48.5-67.1)
		+	24.2 (18.6-26.1)			+	**31.0 (21.0-42.0) * **

**S**	pH	20 µM perifosine	Median of % cell population (min-max)		pH	20 µM perifosine	Median of % cell population (min-max)
normoxia	6.6	-	15.0 (13.4-16.3)	hypoxia	6.6	-	7.2 (3.6-10.3)
		+	15.3 (10.9-20.0)			+	6.5 (5.3-6.9)
	7.3	-	12.5 (8.8-14.9)		7.3	-	11.4 (3.0-24.2)
		+	9.2 (8.6-19.2)			+	6.9 (4.5-13.2)
	7.4	-	12.3 (11.6-13.3)		7.4	-	10.6 (6.0-26.9)
		+	13.3 (9.3-18.3)			+	9.1 (7.4-13.4)

	pH	20 µM perifosine	Median of % cell population (min-max)		pH	20 µM perifosine	Median of % cell population (min-max)
	7.9	-	19.7 (18.1-21.4)		7.9	-	8.9 (4.9-10.7)
		+	15.0 (13.4-16.3)			+	7.8 (7.2-24.6)
**G2/M**	pH	20 µM perifosine	Median of % cell population (min-max)		pH	20 µM perifosine	Median of % cell population (min-max)
normoxia	6.6	-	12.2 (10.9-15.6)	hypoxia	6.6	-	25.5 (16.8-28.1)
		+	29.4 (16.8-30.4)			+	21.1 (13.5-21.3)
	7.3	-	11.2 (9.3-12.7)		7.3	-	22.8 (19.4-30.0)
		+	**10.4 (7.5-15.9) *****			+	**6.1 (4.2-9.7) *****
	7.4	-	10.4 (9.5-12.0)		7.4	-	**13.2 (11.0-14.1) ****
		+	**10.9 (9.3-21.3) *****			+	**5.8 (5.4-8.6) *****
	7.9	-	11.7 (10.5-12.5)		7.9	-	19.9 (15.0-21.3)
		+	**5.8 (4.7-8.6) *****			+	**5.3 (5.0-5.5) *****
